# Magnetic‐Driven Torque‐Induced Electrical Stimulation for Millisecond‐Scale Wireless Neuromodulation

**DOI:** 10.1002/adhm.202500805

**Published:** 2025-06-16

**Authors:** Chao‐Chun Cheng, Li‐Ling Chen, Guan‐Jhong Tseng, Jun‐Xuan Huang, Yen‐Jing Ting, Po‐Han Chiang

**Affiliations:** ^1^ Institute of Biomedical Engineering National Yang Ming Chiao Tung University Hsinchu 30010 Taiwan(R.O.C.)

**Keywords:** barium titanate nanoparticles, magnetite nanodiscs, magnetoelectric stimulation, millisecond‐scale temporal precision, wireless neuromodulation

## Abstract

Wireless neuromodulation using nanoparticles, offering minimally invasive alternatives to conventional deep brain stimulation (DBS) while reducing the risks associated with hardware implants, has gained significant traction over the past decade. Nevertheless, ensuring millisecond‐scale wireless DBS for the precise temporal control of neuronal activity remains challenging. This study reports magnetic‐driven torque‐induced electrical stimulation (MagTIES), a torque‐based magnetoelectric neuromodulation method. By utilizing magnetic nanodiscs to generate torque under alternating magnetic fields (AMFs), the MagTIES induces a piezoelectric effect in piezoelectric nanoparticles, thereby overcoming the limitations of traditional magnetostriction‐based systems. With an AMF (50 mT at ≈10 Hz), the proposed approach triggers neuronal activity both in vitro and in vivo, specifically in the deep brain region of the amygdala, within milliseconds. Furthermore, MagTIES enables the fine‐tuning of amygdala brain oscillations through the precise modulation of the AMF frequency. By combining high spatial and temporal precision with minimal invasiveness, MagTIES provides an innovative approach for advancing neuroscience research with potential applications in understanding neural circuits and developing innovative therapies.

## Graphical abstract

MagTIES is a wireless neuromodulation technique with millisecond precision. It employs magnetic‐driven torque from magnetite nanodiscs to activate piezoelectric nanoparticles, enabling precise temporal control of neuronal activity and brain oscillations using weak, low‐frequency magnetic fields. Free from hardware implants and genetic tools, MagTIES offers a minimally invasive, scalable platform for next‐generation deep brain stimulation.

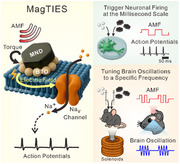

## 1 Introduction

Brain‐activity regulation through electrical stimulation is a pivotal technique in modern neuroscience research and clinical neurological therapy that can enable the treatment of a spectrum of neurological disorders, ranging from Parkinson's disease and epilepsy to chronic pain, affecting millions of people worldwide. Deep brain stimulation (DBS), a prominent treatment method involving electrode implants, has relieved over 160 000 patients with various neurological disorders worldwide over the last 30 years.^[^ ^1^ ^]^ Despite the clinical success of DBS, the electrical implants used in this technique are associated with certain limitations, such as complications from surgery, infection, and potential damage due to micromotions during daily activities.^[^ ^2^ ^]^ Consequently, developing less‐invasive methods of electrical brain stimulation is essential.

To minimize invasiveness, recent advances have shifted toward magnetic approaches^[^ ^3^, ^4^, ^5^, ^6^ ^]^ that utilize the ability of magnetic fields to penetrate the skull, bones, and tissues without interference, thereby enabling the remote manipulation of neuronal activity.^[^ ^5^ ^]^ Transcranial magnetic stimulation (TMS), the most prominent of these methods, uses strong magnetic fields (>1.5 T) with a high rate of change (dB/dt) to non‐invasively induce electrical currents within the brain. However, the spatial and depth precision of TMS is limited by its capacity to stimulate deep brain regions without affecting the surface areas,^[^ ^7^ ^]^ thereby restricting its scope of application. The limitations of these methods have led to the exploration of alternative magnetic‐based neuromodulation strategies that can reach deep neural targets with good spatial precision. Among the investigated techniques, magnetic‐nanoparticle‐based neuromodulation, in which magnetic nanoparticles are remotely manipulated with lower magnitude magnetic fields than those used in TMS, offers a potentially transformative solution for deep brain stimulation.^[^ ^5^, ^8^ ^]^

Over the last decade, various magnetic‐nanoparticle‐based neuromodulation approaches have been invented for wirelessly stimulating deep brain neurons with minimal invasiveness, such as magnetothermal stimulation,^[^ ^8^, ^9^ ^]^ magnetomechanical stimulation,^[^ ^3^, ^4^, ^10^ ^]^ magnetochemogenetics,^[^ ^11^ ^]^ and magnetoelectric stimulation.^[^ ^12^, ^13^, ^14^, ^15^, ^16^, ^17^, ^18^ ^]^ Among these, magnetoelectric stimulation is the only approach that does not require the expression of intrinsic or exogenous thermosensitive and mechanosensitive ion channels or specific receptors in the target neurons. Besides avoiding the complexities associated with gene delivery and exogenous gene overexpression, this approach sidesteps the challenge of variable intrinsic mechanosensor expression, which is poorly understood and complicates the predictability of neuromodulation effects in diverse neuronal types. However, magnetoelectric stimulation is associated with certain limitations.

Millisecond‐scale temporal precision is essential for modulating neuronal activity, as various brain functions, including synaptic transmission, action potential firing, and neural oscillations, operate on this timescale. Brain oscillations are fundamental to processes such as memory, cognition, and decision making,^[^ ^19^, ^20^, ^21^, ^22^ ^]^ and disruptions in these oscillatory patterns are associated with neurological and psychiatric disorders, including epilepsy, Parkinson's disease, depression, and schizophrenia​​.^[^ ^23^, ^24^ ^]^ Current magnetoelectric neuromodulation technologies, which are effective in some contexts, lack the temporal resolution necessary to align stimulation with specific oscillatory phases, limiting their ability to precisely target neural circuits​. Ensuring millisecond‐scale precision in stimulation can enable synchronization with endogenous oscillations, allowing neural‐network modulation with good specificity. Therefore, such a technique can reveal vital information on brain functions and open new avenues in the treatment of oscillation‐related disorders. These considerations underscore the importance of developing new neuromodulation approaches that enable precise temporal control while overcoming the limitations of current magnetoelectric systems.

Current magnetoelectric nanomaterials for neuromodulation predominantly focus on magnetostriction‐based approaches that utilize superparamagnetic and piezoelectric materials.^[^ ^12^, ^13^, ^14^, ^15^, ^16^, ^17^, ^18^ ^]^ The effectiveness of these methods depends largely on the coupling efficiency between the magnetic and piezoelectric elements; core–shell architectures are frequently used in these systems to optimize this interaction. Several studies report spherical and disc‐shaped nanomaterial composites, such as CoFe_2_O_4_–BaTiO_3_ nanoparticles,^[^ ^12^, ^16^ ^]^ CoFe_2_O_4_–BiFeO_3_ nanoparticles,^[^ ^13^, ^14^ ^]^ and Fe_3_O_4_–CoFe_2_O_4_–BaTiO_3_ nanodiscs,^[^ ^17^ ^]^ with high potential applicability in various fields ranging from DBS^[^ ^16^, ^17^ ^]^ and Aβ dissociation^[^ ^13^ ^]^ to tumor‐cell apoptosis induction.^[^ ^14^ ^]^ For effective DBS, these technologies often require a large static magnetic field of ≈220 mT coupled with an alternating magnetic field (AMF) of >6 mT at frequencies beyond 140 Hz.^[^ ^16^, ^17^ ^]^ However, magnetoelectric nanoparticles^[^ ^12^, ^16^ ^]^ and nanodiscs^[^ ^17^ ^]^ offer only second‐scale temporal precision. Therefore, advancement toward millisecond‐scale precision is vital for the precise modulation of neuronal activity.

Magnetite nanodiscs (MNDs), which can transduce magnetic fields into torques,^[^ ^3^, ^4^, ^10^, ^25^ ^]^ have been recently used for magnetoelectric stimulation via the magnetostriction of their ferromagnetic cores.^[^ ^17^ ^]^ However, the prospect of harnessing the torque generated by MNDs for magnetoelectric stimulation remains unexplored. Adapting the MND‐mediated magnetic‐driven torque for magnetoelectric stimulation could lower the requirements of the magnetic field intensity and frequency, enabling the upscaling of magnetic apparatus for broad applications. BaTiO_3_ nanoparticles (BTOs), commonly used piezoelectric nanoparticles with a high piezoelectric coefficient,^[^ ^26^ ^]^ can be used for the ultrasound‐based wireless stimulation of neuron‐like SH‐SY5Y cells.^[^ ^18^ ^]^ However, to date, the potential of utilizing magnetically driven torques to induce a dielectric effect in BTOs remains unclear.

**Figure 1:**
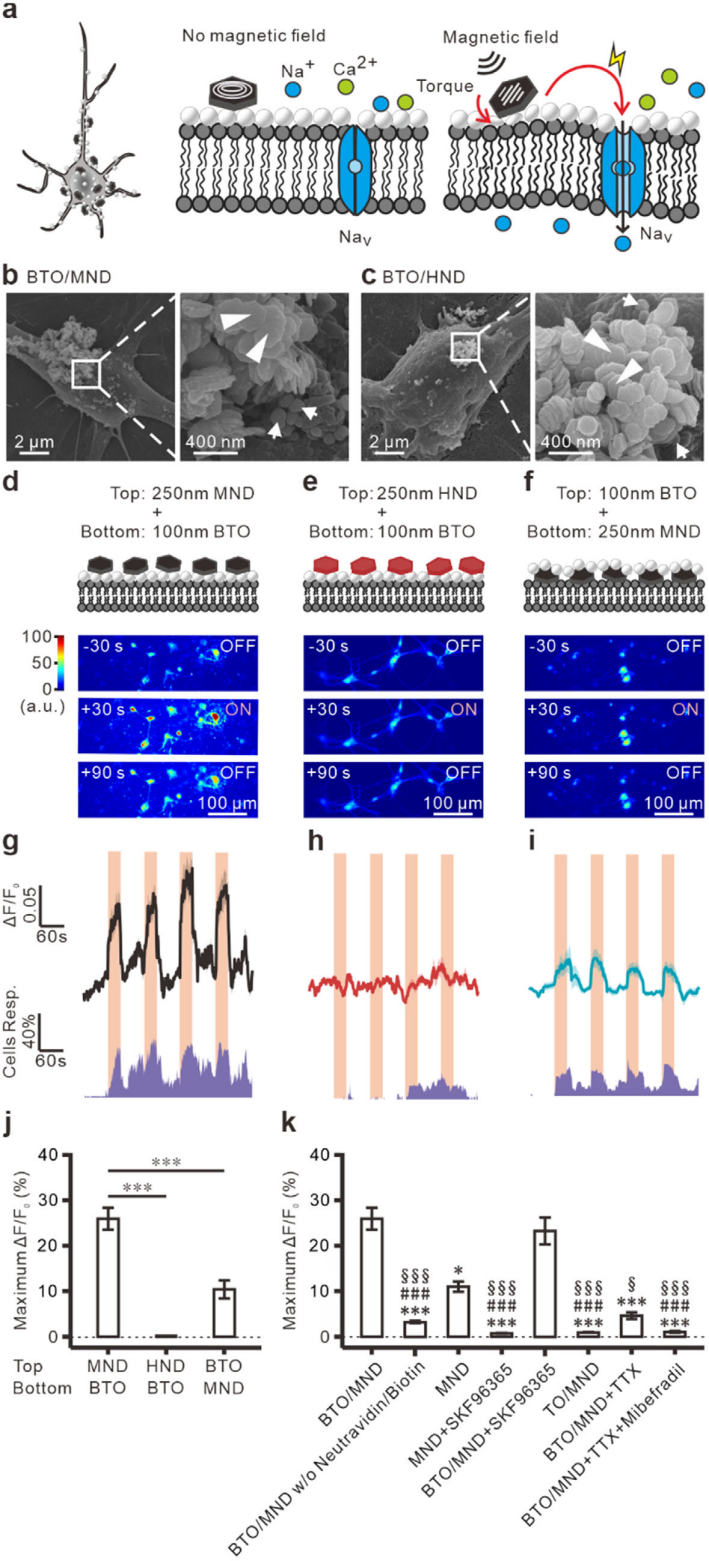
Wireless neuromodulation by MagTIES in vitro. a) Experimental Scheme. Under an external AMF, the torque generated by the MNDs is transduced to the BTOs, triggering piezoelectric responses. Furthermore, the electric field generated by the BTOs can activate the neurons by triggering voltage‐gated channels. b) SEM image of a neuron covered with BTO_100_/MND_250_. c) SEM image of a neuron covered with BTO_100_/HND_250_. d–f) Fluorescence change in the different experimental groups. Top: Schematics of nanomaterial arrangement in the BTO_100_/MND_250_ (d), BTO_100_/HND_250_ (e), and MND_250_/BTO_100_ (f) groups. Bottom: Color maps of the fluorescence intensity in neurons before, during, and after AMF stimulation. g–i) Fluorescence changes (top) and cell response rates (bottom) in the BTO_100_/MND_250_ (g), BTO_100_/HND_250_ (h), and MND_250_/BTO_100_ (i) groups under an AMF (light orange; 50 mT, 10 Hz). j) Maximum fluorescence change (the largest Δ*F*/*F*_0_ during and after the stimulation periods) in the BTO_100_/MND_250_ (*n* = 139, sample = 12), BTO_100_/HND_250_ (*n* = 34, sample = 6), and MND_250_/BTO_100_ (*n *= 53, sample = 6) groups, ***p < 0.001. Tukey post‐hoc test after one‐way ANOVA. k) Maximum fluorescence change of neurons under different conditions: *p = 0.014, ***p < 0.001, compared with the BTO_100_/MND_250_ group (*n *= 139, sample = 12); ###*p *< 0.001, compared with the only MND group; and §*p* = 0.02, §§§*p* < 0.001, compared with the BTO_100_/MND_250_ + SKF96365 group. Tukey post‐hoc test after one‐way ANOVA.

This study proposes an innovative magnetoelectrical neuromodulation technique based on this concept, magnetic‐driven torque‐induced electric stimulation (MagTIES), which uses uniquely configured piezoelectric BTOs and MNDs. By sequentially applying these nanoparticles to cells or tissues and conjugating them via biotin–avidin linkage, the torques generated by MNDs under an AMF are proposed to induce mechanical stress and strain on BTOs strategically positioned between the MNDs and cell membranes (**Figure** **1**a). This mechanical stress/strain leads to the generation of localized electric fields by BTOs through the piezoelectric effect. These electric fields are hypothesized to activate voltage‐gated ion channels on nearby cell membranes, thereby eliciting action potentials in neurons. Compared with previously reported magnetoelectric stimulation methods, this innovative approach requires a lower magnetic density (50 mT) and frequency (≈10 Hz), potentially offering a more precise, less invasive, and deeper‐reaching neuromodulation technique. MagTIES, which combines millisecond‐scale precision with reduced magnetic field requirements, represents a significant advancement in neuromodulation technologies that addresses the limitations of existing methods in this field.

## 2 Results

### 2.1 Nanomaterial Synthesis

Here, MNDs were synthesized using a previously described two‐step solvothermal procedure^[^ ^3^, ^4^ ^]^ and characterized using transmission electron microscopy (TEM), X‐ray diffraction (XRD), and vibrating‐sample magnetometry (Figure S1a–d, Supporting Information). In addition, BTOs (US Research Nanomaterials, Inc.) were characterized by TEM and XRD (Figure S1e,f, Supporting Information). All the nanomaterials were functionalized for cellular applications; the BTOs were coated with poly(ethylene glycol) (PEG) to facilitate attachment with cell membranes^[^ ^18^ ^]^ and functionalized with neutravidin (Figure S1g, Supporting Information). As reported previously,^[^ ^3^, ^4^ ^]^ both the MNDs and hematite nanodiscs (HNDs) were coated with poly(maleic anhydride‐alt‐1‐octadecene) (PMAO) (Figure S1h, Supporting Information); these nanodiscs were then conjugated with biotin. The biotin–avidin linkage, one of the strongest non‐covalent interactions in nature,^[^ ^27^ ^]^ was utilized to bind these nanodiscs to the BTOs.

Notably, a permanent magnet can remove both MNDs and BTOs from a solution containing a mixture of biotinylated 250‐nm MNDs (MND_250_) and neutravidin‐conjugated 100‐nm BTOs (BTO_100_) (Figure S2a, Supporting Information). In contrast, a permanent magnet can only remove the MNDs from a solution containing a mixture of MND_250_ and BTO_100_ without neutravidin/biotin (Figure S2b, Supporting Information). These results were confirmed by measuring the concentrations of the remaining Ba, Ti, and Fe using inductively coupled plasma analysis (Figure S2c–e, Supporting Information). Moreover, the binding of nanomaterials to primary cultured cells was characterized using Förster resonance energy transfer (FRET) analysis. BTO_100_ and MND_250_ were conjugated to Alexa‐488 and ‐594, respectively. Alexa‐488‐conjugated BTO_100_ was applied to primary cultured hippocampal neurons, into which Alexa‐594‐conjugated MND_250_ was subsequently added. When BTO is linked with MND, the Alexa‐594 on MND_250_ emits red fluorescence upon receiving energy from the nearby green fluorescence of Alexa‐488 on BTO_100_ during blue light application (Figure S2f, Supporting Information). The FRET ratio increases significantly after the application of MNDs, confirming their binding (Figure S2g,h, Supporting Information). In contrast, the FRET ratio remains unchanged when fluorophore‐conjugated nanomaterials are used without a biotin–neutravidin linkage. (Figure S2i,j, Supporting Information). These results indicate that the distance between MND and BTO is less than the Förster distance (10 to 100 Å)^[^ ^28^ ^]^ for the biotin–neutravidin linkage. Scanning electron microscopy (SEM) and energy dispersive spectroscopy (EDS)–SEM images of neurons in the BTO_100_/MND_250_ group show that BTO_100_ is attached to the cell membrane, and MND_250_ is attached to BTO_100_ on the membrane (Figure 1b; Figure S3a, Supporting Information). Similar results are observed in the SEM and EDS–SEM images of the neurons treated with BTO_100_ and 250‐nm HNDs (HND_250_) (Figure 1c; Figure S3b, Supporting Information).

### 2.2 MagTIES‐Induced Neuronal Responses In Vitro

In this study, the magnetoelectric‐stimulated Ca^2+^ responses were measured in cultured neurons (Figure 1d–j; Figure S4, Supporting Information). On sequentially treating neurons with functionalized BTO_100_ and MND_250_, magnetic‐induced Ca^2+^ responses are observed during the application of slow and weak AMF (50 mT at 10 Hz; Figure 1d). Repeated AMF exposure at this intensity and frequency elicits multiple Ca^2+^ responses (Figure 1g), which are not observed in the control group treated with BTO_100_/HND_250_ (Figure 1e,h). Moreover, the response intensity is significantly higher in the BTO_100_/MND_250_ group (Figure 1j), confirming the specificity of the MagTIES‐mediated responses. Interestingly, treating neurons with MND_250_ before BTO_100_ results in significantly smaller AMF‐induced responses (Figure 1f,i,j). These results indicate that the arrangement of BTOs between MNDs and cell membranes is critical for inducing neuronal activity. Notably, on using BTO_100_/MND_250_ without neutravidin/biotin, the magnetic field does not induce any Ca^2+^ response, indicating that the biotin–neutravidin linkage between the BTOs and MNDs is critical for MagTIES (Figure 1k; Figure S5a, Supporting Information).

According to a previously published study, the intrinsic mechanosensitive ion channel transient receptor potential canonical (TRPC) channels in hippocampal neurons^[^ ^4^ ^]^ can be activated by magnetomechanical stimulation with MND_250_ alone.^[^ ^4^ ^]^ In this study, the MagTIES‐induced responses are significantly greater than those caused by MND_250_ alone (Figure 1k; Figure S5b, Supporting Information). Interestingly, applying the TRPC‐specific antagonist SKF96365 significantly reduces Ca^2+^ responses in the MND_250_‐only group (Figure 1k; Figure S5c, Supporting Information) without reducing the MagTIES‐induced neuronal activity with BTO_100_/MND_250_ (Figure 1k; Figure S5d, Supporting Information). Moreover, no response is observed when a layer of non‐piezoelectric nanoparticles, 100‐nm neutravidin‐conjugated PEGylated TO_2_ nanoparticles (TO_100_), is applied between MND_250_ and the membrane (Figure 1k; Figure S5e, Supporting Information). These results indicate that MagTIES‐induced activity is independent of intrinsic mechanosensitive channels. Additionally, the antagonists of voltage‐gated Na^+^ and Ca^2+^ channels, tetrodotoxin (TTX) and mibefradil, respectively, were used to confirm the dependence of MagTIES‐induced Ca^2+^ responses on voltage‐gated channels. Both voltage‐gated channel blockers abolish MagTIES‐induced responses (Figure 1k; Figure S5f–h, Supporting Information).

In addition, the biosafety and cellular interactions of the nanoparticles in cultured neurons were investigated. The propidium iodide (PI) uptake assay^[^ ^29^ ^]^ indicates minimal cytotoxicity in both the BTO_100_/MND_250_ and BTO_100_/HND_250_ groups after AMF application (Figure S6a–c, Supporting Information), suggesting good biocompatibility under the experimental conditions. To investigate the possibility of nanoparticle internalization, neurons were analyzed with TEM after 0–7 days of incubation with BTO_100_/MND_250_. No noticeable uptake of BTO_100_ and MND_250_ is shown by the cultured neurons (Figure S6d–f, Supporting Information), suggesting that these nanoparticles remain extracellular and stable throughout the experimental period. Similarly, the live/dead (Calcein‐AM/PI) assay in HEK293T cells indicates low levels of cell death in both treatment groups following AMF exposure (Figure S6d–f, Supporting Information). Collectively, these results support the biosafety and in vitro stability of the MagTIES system using a combination of BTO_100_ and MND_250_.

### 2.3 MagTIES with Nanomaterials in Different Sizes

**Figure 2:**
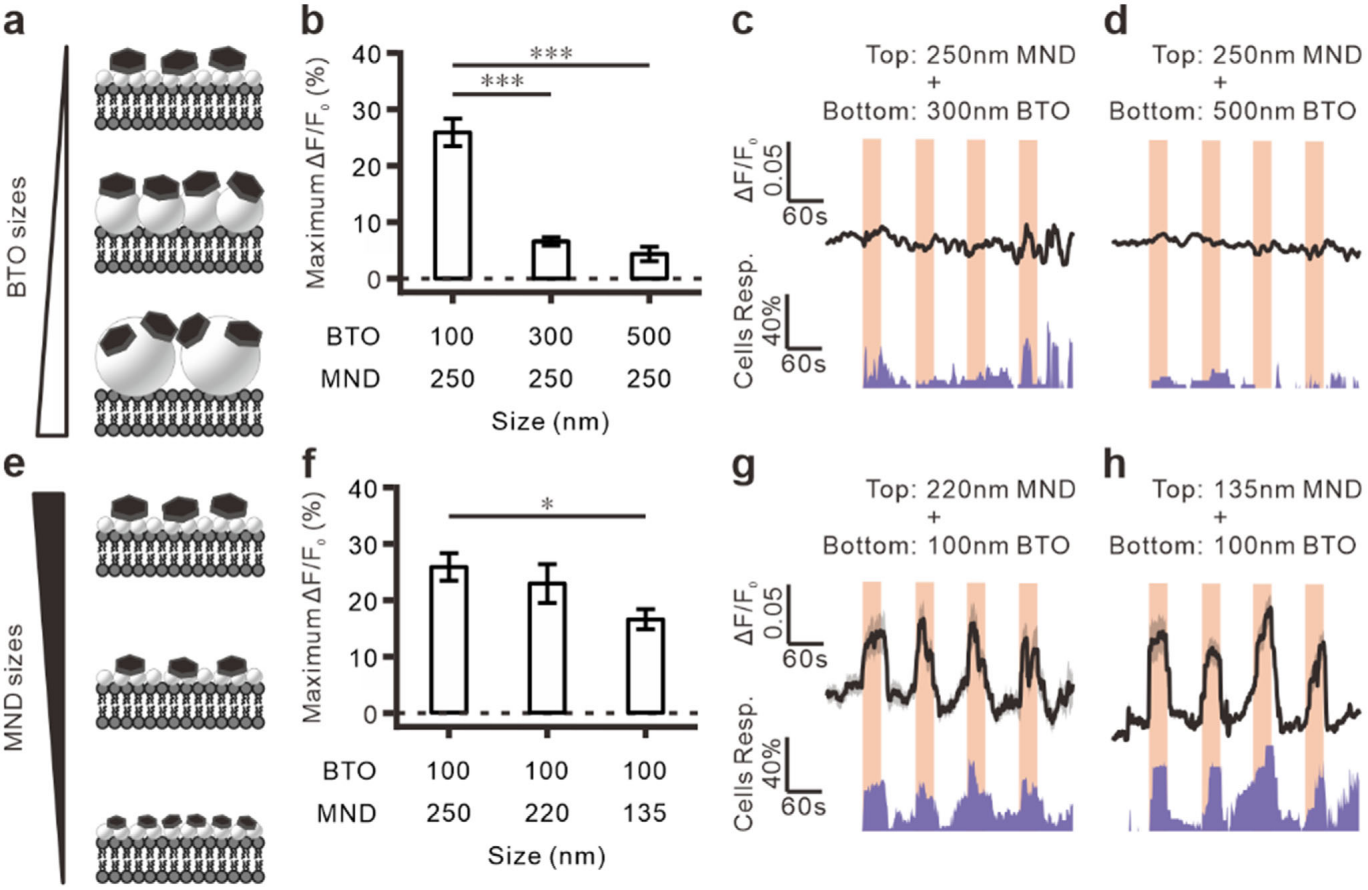
MagTIES with nanomaterials in different sizes. a) Schematics of BTOs/MNDs combination with different sizes of BTOs with MND_250_. b) Maximum fluorescence change in neurons with MND_250_ and different sizes of BTOs (BTO_100_/MND_250_, *n* = 139, sample = 12; BTO_300_/MND_250_, *n* = 34, sample = 6; BTO_500_/MND_250_, *n* = 34, sample = 6). ***p < 0.001, Tukey post‐hoc test after one‐way ANOVA. c,d) Calcium responses in the BTO_300_/MND_250_ (c, *n* = 34, sample = 6) and BTO_500_/MND_250_ (d, *n* = 34, sample = 6) groups under an AMF (50 mT, 10 Hz). Top: Averaged fluorescence changes. Bottom: Cell response rates. Light orange areas indicate periods of AMF application. e) Schematics of BTOs/MNDs combination with different sizes of MNDs with BTO_100_. f) Maximum fluorescence changes in neurons with BTO_100_ and different sizes of MNDs (BTO_100_/MND_250_, same as (a); BTO_100_/MND_220_, *n *= 50, sample = 6; BTO_100_/MND_135_, *n* = 76, sample = 9). *p = 0.025, Tukey post‐hoc test after one‐way ANOVA. g,h) Calcium responses in the BTO_100_/MND_220_ (g, *n* = 50, sample = 6) and BTO_100_/MND_135_ (h, *n* = 76, sample = 9) groups under an AMF (50 mT, 10 Hz). Top: Averaged fluorescence changes. Bottom: Cell response rates. Light orange areas indicate periods of AMF application.

Although macroscale tetragonal BTO typically shows a higher piezoelectric coefficient, a previous study reports that near‐cubic BTO nanoparticles (<120 nm) show strong piezoelectricity with *d33 > *1500 pC N^−1^.^[^ ^26^ ^]^ These strong piezoelectric properties may be attributed to topological vortex structures at the nanoscale,^[^ ^30^ ^]^ which create regions of high flexibility that enhance the piezoelectric response by enabling large atomic displacements.^[^ ^30^ ^]^ As the size increases to 300–1000 nm, the piezoelectric coefficient decreases to <300 pC N^−1^, approaching bulk‐material behavior.^[^ ^26^, ^30^ ^]^ Therefore, MND_250_ with BTOs of different sizes: 100 nm (BTO_100_), 300 nm (BTO_300_), and 500 nm (BTO_500_) were used for analysis in this study (**Figure** **2**a; Figure S7a–c, Supporting Information). Ca^2+^ imaging indicates that BTO_100_/MND_250_ elicits significantly stronger neuronal responses than BTO_300_/MND_250_ and BTO_500_/MND_250_ (Figure 2b–d), suggesting that the stimulated response is inversely correlated with the BTO size.

Here, the torque generated by the MNDs was hypothesized to be positively correlated with the stress/strain on the BTO and the piezoelectric responses. According to previous studies, the size of nanodiscs positively correlates with the MND‐mediated torque^[^ ^3^, ^4^ ^]^ In this study, the MagTIES‐induced neuronal responses to BTO_100_ with MND_250_, 220‐nm MNDs (MND_220_), and 135‐nm MNDs (MND_135_) were compared (Figure 2e; Figure S7d–f, Supporting Information). The BTO_100_/MND_250_ group shows a significantly stronger response than the BTO_100_/MND_135_ group and a slightly more robust response than the BTO_100_/MND_220_ group (Figure 2f–h). These results indicate that MagTIES with larger MNDs can induce more effective responses.

### 2.4 MagTIES‐Induced Action Potentials at the Millisecond Scale

**Figure 3:**
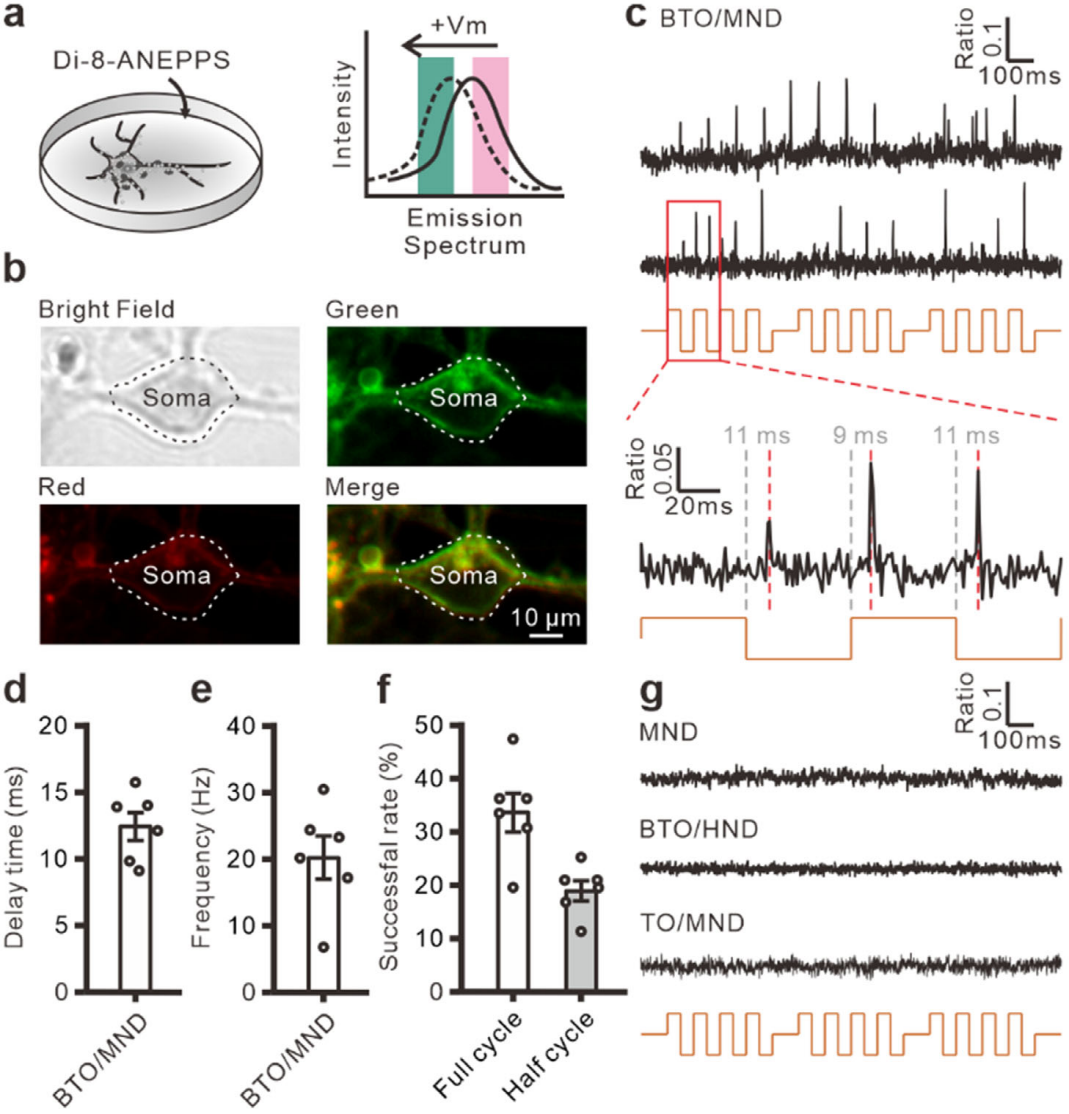
MagTIES‐induced action potentials in the cultured neuron. a) Schematics of the emission spectrum shift of Di‐8‐ANEPPS on increasing the membrane potential (+*V*_m_). b) Green (top), red (middle), and merged (bottom) fluorescence image for a neuron with Di‐ANEPPS. c) MagTIES‐induced voltage responses in the BTO_100_/MND_250_ group. Top: Traces of the green/red ratio of Di‐8‐ANEPPS from two individual neurons. Bottom: Enlargement of a trace from the top. d) Averaged delay time of action potentials from individual neurons (*n* = 6, sample = 6). e) Averaged action potential frequencies from individual neurons (*n* = 6, sample = 6). f) Success rate (%) calculated as the percentage of full or half cycles of AMF stimulation that induce at least one detectable action potential. g) Representative voltage imaging traces from control groups (MND_250_‐only, BTO_100_/HND_250_, and TO_100_/MND_250_) under identical stimulation conditions showing no detectable membrane potential changes.

In Ca^2+^ imaging, MagTIES induces immediate Ca^2+^ responses when an AMF is applied (Figure 1k). In contrast, magnetomechanical‐stimulated Ca^2+^ responses by MND_250_ alone are generated significantly slower on AMF application (Figure S5b,c, Supporting Information), consistent with a previous report on magnetomechanical stimulation with MNDs.^[^ ^4^ ^]^ To elucidate the temporal precision of MagTIES further, Di‐8‐ANEPPS, a ratiometric voltage‐sensitive dye, was used to measure the action potentials in the cultured neurons at a temporal resolution of 996 Hz. Di‐8‐ANEPPS shifts its emission spectrum upon neuronal depolarization when excited at 470 nm, enabling the quantification of membrane potential changes through a comparison of the green and red emissions (**Figure** **3**a).^[^ ^31^, ^32^ ^]^ Fluorescence imaging indicates that Di‐8‐ANEPPS is predominantly localized in the plasma membrane (Figure 3b; Figure S8a, Supporting Information), confirming the accuracy of the potential change measurements. Experimentation with a voltage clamp in whole‐cell patch‐clamp electrophysiology indicates that the ratio changes in Di‐8‐ANEPPS can be correlated with the membrane potential (Figure S8b–e, Supporting Information).

By applying short AMF pulses of 400 ms at 50 mT and 10 Hz, MagTIES‐induced neuronal spikes are observed within milliseconds (Figure 3c). Each AMF cycle alternates the external magnetic field between the two directions. Each bidirectional alternation in a cycle generates two distinct torques via MNDs. Consequently, action potentials can be triggered by magnetic‐field alternations in both directions. The delay of controlling signal to the peak of action potential is 12.3 ± 1 ms (Figure 3d), the average frequency of the induced action potentials is 20.2 ± 3.3 Hz (Figure 3e), and the success rate of action‐potential generation in full and half cycles is 33.8 ± 3.7% and 19 ± 1.9%, respectively (Figure 3f). Notably, short AMF pulses do not induce action potentials in the control groups, such as those comprising cells treated with MND_250_ alone, BTO_100_/HND_250,_ and TO_100_/MND_250_ (Figure 3g).

### 2.5 MagTIES‐Induced Neuronal Activity In Vivo

**Figure 4:**
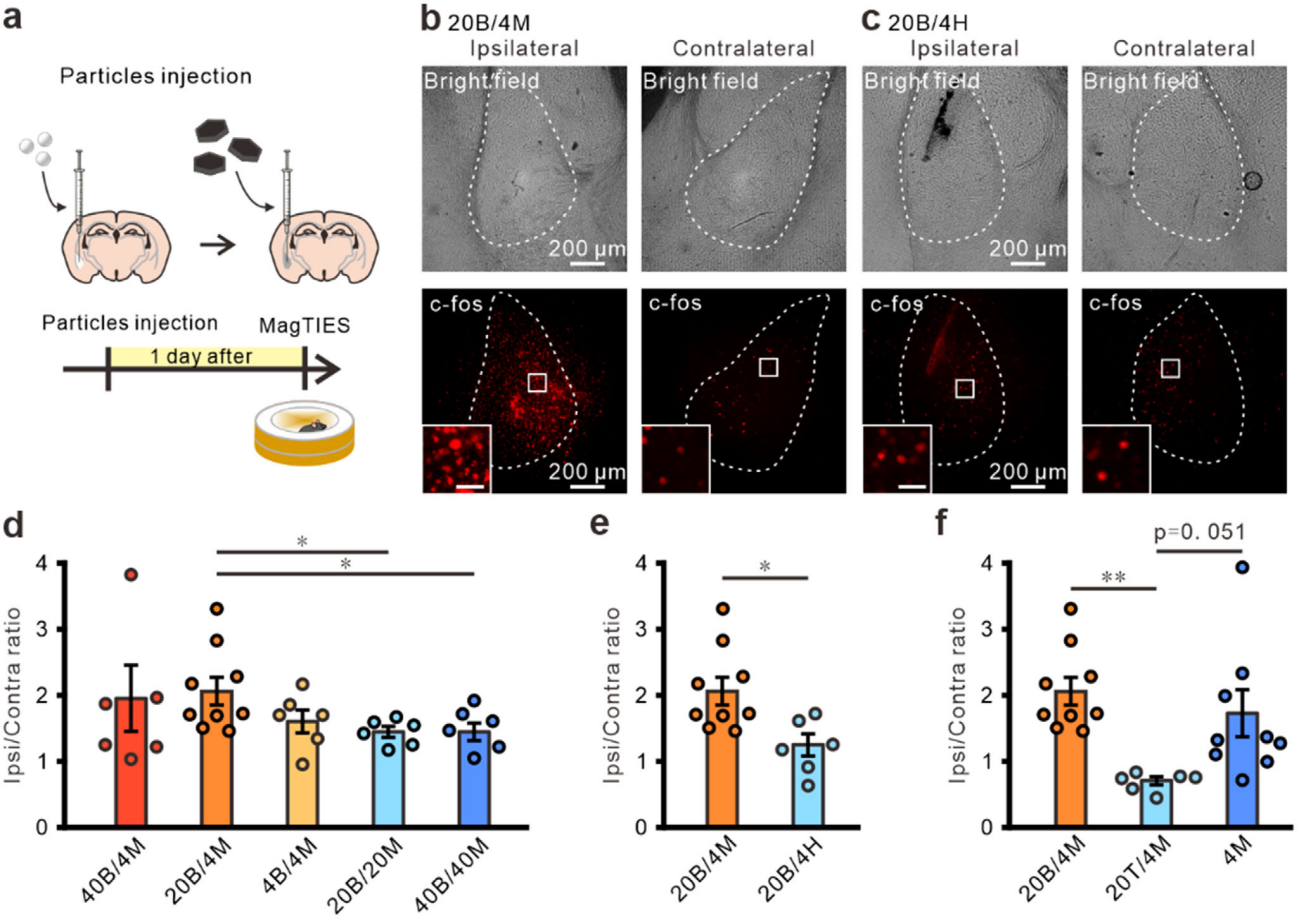
Neuronal responses in vivo by MagTIES with different amounts of BTOs and MNDs. a) Experimental scheme for MagTIES analysis by immunostaining c‐fos. During the MagTIES procedure, an AMF (50 mT at 10 Hz) is applied ten times at 30 s intervals. b,c) Amygdala slices from the 20B/4M (b) and 20B/4H (c) groups. Bright‐field (top) and c‐fos staining (bottom) images of the ipsilateral (left) and contralateral (right) BLA. Inset: Magnified c‐fos image. d) The c‐fos expression level of BLA in the 40B/4M (*n* = 6), 20B/4M (*n *= 9), 4B/4M (*n* = 6), 20B/20M (*n* = 6), and 40B/40M (*n* = 6) groups. *p = 0.023 for 20B/4M vs 20B/20M; *p* = 0.027 for 20B/4M vs 40B/40M, Tukey post‐hoc test after one‐way ANOVA. e) The c‐fos expression level in the 20B/4M (same as d) and 20B/4H groups (*n* = 6). *p = 0.017, unpaired *t*‐test. f) The c‐fos expression level in the 20B/4M (same as d), 20T/4M (*n* = 6), and 4M (*n* = 9) groups. **p = 0.009, Tukey post‐hoc test after one‐way ANOVA.

The amygdala, a crucial area for emotion processing located deep within the brain, was used to investigate the in vivo efficacy of MagTIES, particularly its ability to target deep brain regions.^[^ ^33^, ^34^ ^]^ For the in vivo study, BTO or TO was stereotactically injected into the amygdala before the injection of MNDs or HNDs (**Figure** **4**a). Fluorophore‐labeled nanoparticles were used to confirm in vivo attachment between BTO and the MNDs. Alexa‐488‐conjugated BTO100 was injected into the amygdala before Alexa‐594‐conjugated MND250. The 450 nm excitation of Alexa‐488‐labeled BTOs results in red fluorescence from Alexa‐594‐labeled MNDs, suggesting that the two nanoparticles are in close proximity to brain tissue (Figure S9a, Supporting Information). This observation is consistent with the in vitro data acquired in this study (Figure S2f–j, Supporting Information).

Next, the biocompatibility of the BTOs and MNDs was evaluated using the markers for microglia, ionized calcium‐binding adapter molecule 1 (Iba1) and cluster of differentiation 68 (CD68), and a marker for astrocytes, glial fibrillary acidic protein (GFAP), after 3 days of the stereotaxic injection of BTO_100_/MND_250_ (Figure S9b,e,h, Supporting Information). The expression levels of Iba1, CD68, and GFAP in the BTO_100_/MND_250_ group are similar to those in the control group injected with PBS (Figure S9c–j, Supporting Information), confirming the high biocompatibility of the nanomaterials.

The in vivo neuronal activity induced by MagTIES was investigated by immunostaining for c‐fos, an immediate early gene. Following a day of recovery, mice were exposed to MagTIES within the 11 cm inner‐diameter coils designed to generate an AMF tailored explicitly for this study (Figure 4a; Figure S10, Supporting Information). The mice were acclimated to the chamber (10 cm in diameter and 9 cm in height) for 30 min before stimulation through a protocol comprising 10–30‐s stimulation periods interspersed with 30‐s rest intervals with an AMF of 50 mT at 10 Hz.

To systematically evaluate the effect of MagTIES on neuronal activity, the ratios and amounts of the injected BTOs and MNDs were varied; for example, 40 µg BTOs/4 µg MNDs (40B/4M), 20 µg BTOs/4 µg MNDs (20B/4M), and 4 µg BTOs/4 µg MNDs (4B/4M). Additionally, experiments were conducted with different total amounts at the same ratio, including combinations of 20 µg BTOs/20 µg MNDs (20B/20M) and 40 µg BTOs/40 µg MNDs (40B/40M). Before craniotomy, functionalized BTOs and MNDs were characterized by FRET in vitro to confirm the function of the biotin–avidin linkage in each batch of materials (Figure S2, Supporting Information). Among these groups, the c‐fos expression ratio (ipsilateral/contralateral) of the 20B/4M group is significantly higher than those of the 20B/20M and 40B/40M groups (Figure 4b,d; Figure S11a–e, Supporting Information). However, no significant differences are observed among the 40B/4M, 20B/4M, and 4B/4M groups. Next, the 20B/4M group is compared with the negative control group comprising cells treated with 20 µg BTO and 4 µg HNDs (20B/4H) (Figure 4b,c; Figure S11b,f, Supporting Information); the c‐fos expression ratio is significantly higher in the 20B/4M group (Figure 4e). The streptavidin–fluorescein isothiocyanate (FITC) staining of brain slices from the 20B/4M group was used to assess the spatial localization of nanoparticles in vivo. Within the amygdala, biotinylated MNDs are localized near regions showing MagTIES‐induced c‐fos expression (Figure S11i,j, Supporting Information), suggesting spatial proximity between the presence of nanoparticles and neural activation.

In the in vitro study, compared with the BTO/MND group, magnetomechanical stimulation with MNDs triggers much slower neuronal activity (Figure 1k; Figure S5b, Supporting Information). Similar to a previous report,^[^ ^4^ ^]^ MNDs alone increase ipsilateral c‐fos levels (Figure 4f; Figure S11h, Supporting Information). However, when TO_100_ is stereotactically injected before MND_250_, the c‐fos ratio is observed to be significantly lower than that in the BTO_100_/MND_250_ and MND_250_ groups (Figure 4f; Figure S11a–h, Supporting Information), consistent with the Ca^2+^ imaging results of in vitro experiments (Figure 1k; Figure S5e, Supporting Information).

### 2.6 Modulating Neural Oscillation by MagTIES

Brain oscillations in different regions of the brain are crucial for various functions^[^ ^19^, ^20^, ^21^, ^22^ ^]^ Neuromodulation technologies with high temporal precision, such as electrical DBS and optogenetics, can target and modulate oscillations at specific frequencies,^[^ ^21^, ^22^, ^35^ ^]^ enabling the manipulation of neuronal circuitry by controlling neuronal activity with precise timing. Beta‐band oscillations (≈13–30 Hz) are functionally significant in the amygdala for encoding emotional valence and guiding reward‐based decision‐making.^[^ ^36^, ^37^ ^]^ Optogenetic studies using stimulation at ≈20 Hz support the behavioral importance of beta‐frequency activity in amygdala circuits, showing its capacity to modulate anxiety, reinforce reward seeking, and enhance motivational salience.^[^ ^38^, ^39^, ^40^ ^]^

**Figure 5:**
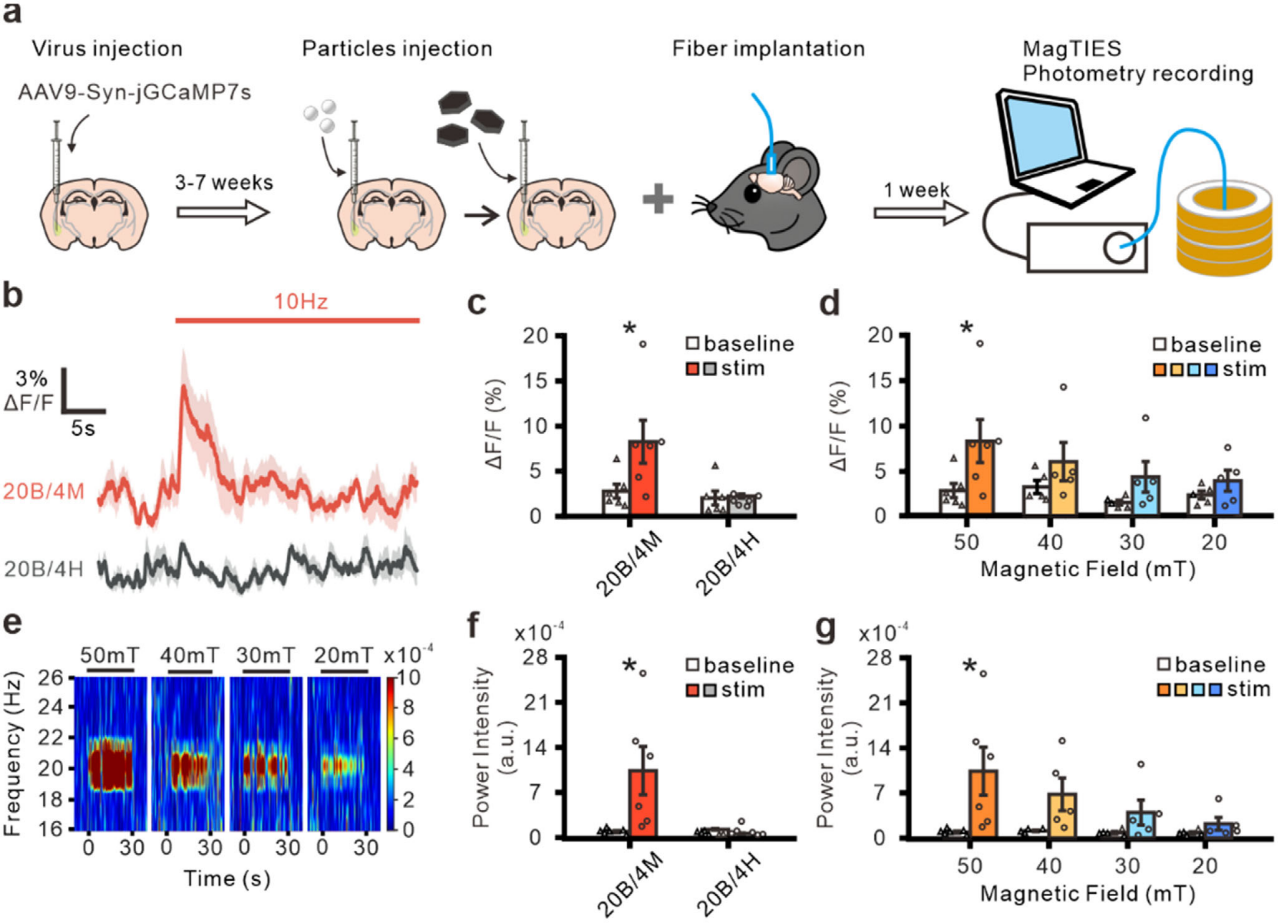
MagTIES induced Ca2+ response in vivo. a) Experimental scheme for MagTIES analysis by fiber photometry b) Average trace of Δ*F*/*F* in the 20B/4M (top, *n* = 6) and 20B/4H (bottom, *n* = 6) groups. The bar indicates the period when an AMF (50 mT, 10 Hz) is applied. c) Averaged Δ*F*/*F* before and during stimulation with the AMF (50 mT, 10 Hz). (20B/4M, *n* = 6; 20B/4H, *n* = 6). *p = 0.024, paired *t*‐test. d) Averaged Δ*F*/*F* from mice in the 20B/4M group before and during stimulation with different intensities of an AMF (10 Hz). (50 mT, *n* = 6; others, *n *= 5). *p = 0.024, paired *t*‐test. e) Power spectrum of a 10‐Hz AMF at different intensities from a mouse in the 20B/4M group. The stimulation periods are indicated by the bar. f) Averaged power intensity before and during AMF (50 mT, 10 Hz) stimulation recorded in the 20B/4M (*n* = 6) and 20B/4H (*n* = 6) groups. *p = 0.049, paired *t*‐test. g) Averaged power intensity before and during stimulation with 10‐Hz AMF at different intensities in the 20B/4M group. (50 mT, *n* = 6; others, *n *= 5). *p = 0.049, paired *t*‐test.

However, tuning the frequency of brain oscillations using magnetic DBS has not been reported to date. In this study, fiber photometry was used to record real‐time neuronal activity in vivo,^[^ ^41^ ^]^ circumventing the interference of magnetic stimulation in measurements (**Figure** **5**a). AAV‐hSyn‐jGCaMP7s‐WPRE was unilaterally injected into the basolateral amygdala (BLA). After 3 to 7 weeks, 20 µg BTOs and 4 µg MNDs (20B/4M) or 20 µg BTOs and 4 µg HNDs (20B/4H) were stereotaxic injected into the same location, and an optical fiber with 400 µm diameter was implanted into the BLA. Post‐surgical recovery and Ca^2+^ responses in vivo were measured by fiber photometry in a magnetic apparatus with a diameter of 10 cm and height of 19 cm (Figure S10e–g, Supporting Information).

First, MagTIES was carried out using an AMF of 50 mT at 10 Hz for 30 s. In the 20B/4M group, a significant increase is observed in the fluorescence change (dF/F) compared with pre‐stimulation levels (Figure 5b–d). In contrast, no notable change is observed in the fluorescence in the control group treated with 20B/4H (Figure 5b–d). In the 20B/4M group, reducing the magnetic field intensity to 40, 30, and 20 mT results in an observable (but not statistically significant) increase in fluorescence (Figure 5d). Interestingly, fast Fourier transform (FFT) analysis indicates increased brain oscillations at 20 Hz under 10‐Hz AMF in the 20B/4M group (Figure 5e), a phenomenon not observed in the 20B/4H group. Under an AMF of 50mT, the power spectrum intensity at 20 Hz significantly increases for the 20B/4M‐injected mice (Figure 5f); this phenomenon is not observed in the 20B/4H‐injected mice. Moreover, the power spectrum intensity increases notably (but without statistical significance) when the magnetic field intensity is reduced to 40, 30, and 20 mT (Figure 5g).

**Figure 6:**
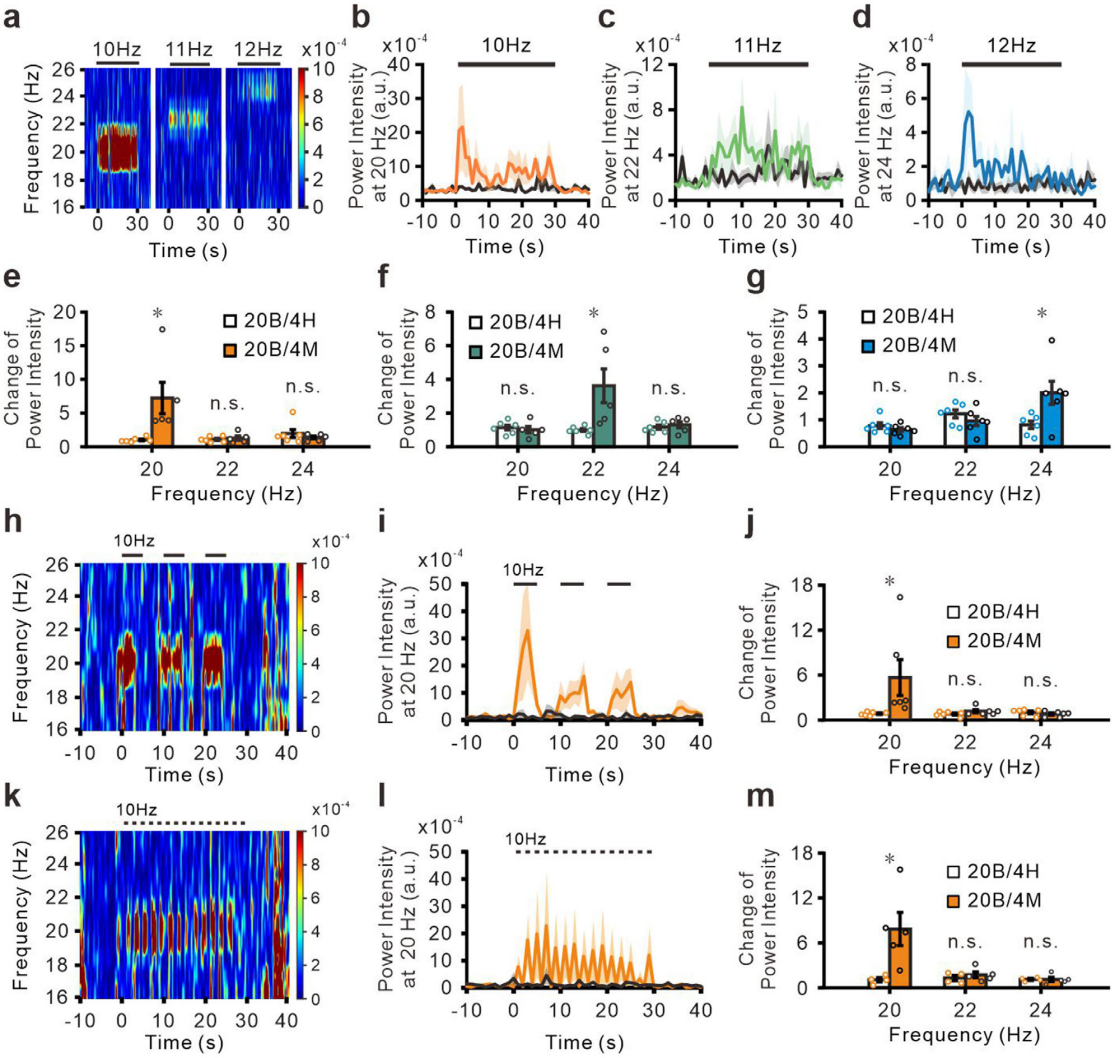
Controlling brain oscillation by MagTIES with high temporal precision. a) Power spectrum of MagTIES with 50‐mT AMF at 10, 11, and 12 Hz for 30 s. b–d) Averaged traces of power intensity at 20 (b), 22 (c), and 24 Hz (d) are obtained using MagTIES with 10‐ (b), 11‐ (c), and 12‐Hz (d) AMF stimulations, respectively. Orange (b), green (c), and blue (d) traces are recorded in the 20B/4M group (*n* = 5). Black traces are from the 20B/4H group (*n* = 6). e–g) Change in power intensity at 20, 22, and 24 Hz from the 20B/4M (*n* = 5) and 20B/4H (*n* = 6) groups is observed on MagTIES with 10‐Hz (e; *p = 0.027, unpaired *t*‐test), 11‐Hz (f; *p = 0.03, unpaired *t*‐test), and 12‐Hz (g; *p = 0.037, unpaired *t*‐test) AMF stimulation. h) Power spectrum of MagTIES with 5‐s AMF at 10 Hz (thrice) with 5‐s intervals. i) Averaged traces of power intensity at 20 Hz under the same conditions as (h). Orange represents data from the 20B/4M group (*n* = 4). Black represents data from the 20B/4H group (*n* = 6). j) Change of power intensity at 20, 22, and 24 Hz from the 20B/4M and 20B/4H groups under the same condition as (h). *p = 0.036, unpaired *t*‐test k) Power spectrum of MagTIES with 1‐s AMF at 10 Hz (15 times) with 1‐s intervals. l) Averaged traces of the power intensity at 20 Hz under the same condition as (k). Orange represents data from the 20B/4M group (*n* = 5). Black represents data from the 20B/4H group (*n* = 4). m) Change of power intensity at 20, 22, and 24 Hz from the 20B/4M (*n* = 5) and 20B/4H (*n* = 4) groups under the same condition as (k). *p = 0.016, unpaired *t‐*test.

To determine whether the oscillation frequency corresponded with the MagTIES frequency, different AMF frequencies were used for MagTIES. Increasing the AMF frequency from 10 to 11 and 12 Hz results in an apparent increment in the power intensity at 20, 22, and 24 Hz, respectively (**Figure** **6**a–d; Figure S12a–d, Supporting Information). However, no apparent changes are observed outside of the beta band. As hypothesized, the oscillation of Ca^2+^ responses is precisely twice the frequency of the AMF. Under a 10 Hz AMF, the change in the power intensity at 20 Hz in the 20B/4M group is approx. sevenfold more prominent than the baseline intensity (Figure 6e), which is significantly higher than the power‐intensity change observed in the 20B/4H group (Figure 6b,e; Figure S12e, Supporting Information). However, the shift in the power intensity at nearby frequencies is similar for the 20B/4M and 20B/4H groups (Figure 6e). Similarly, using MagTIES with 11 Hz and an AMF of 50 mT, the increase in the power spectrum intensity at 22 Hz is significantly larger in the 20B/4M group than in the 20B/4H group (Figure 6c,f; Figure S12e, Supporting Information). Interestingly, the power intensity does not change at 20 or 24 Hz (Figure 6f). Similar results are observed using MagTIES at 12 Hz and an AMF of 50 mT. Only a change in the power intensity at 24 Hz is observed in the 20B/4M group compared with the 20B/4H group (Figure 6d,g; Figure S12e, Supporting Information), which is not observed at other frequencies (Figure 6g).

Finally, MagTIES‐induced responses were observed, even when the AMF stimulation period was reduced to 5 or 1 s. Specifically, applying AMF for a 5‐s stimulation period interspersed with 5‐s intervals for three cycles leads to a large increase in the power spectrum intensity, which is particularly significant at 20 Hz, during the stimulation periods (Figure 6h,i). The change in power intensity at 20 Hz observed 5 s before and after stimulation is significantly greater in the 20B/4M group than in the 20B/4H group (Figure 6j; Figure S12f, Supporting Information). A similar pattern emerges when the AMF is applied for 1‐s periods followed by 1‐s resting periods for 15 cycles. A significant increase in the power spectrum intensity at 20 Hz is observed during the 1‐s stimulation periods (Figure 6k,l). Notably, the increase in power intensity at 20 Hz is particularly significant in the 20B/4M group (Figure 6m; Figure S12g, Supporting Information). These fiber photometry results confirm the unique capability of MagTIES to manipulate neural oscillations in the amygdala with a high degree of temporal and frequency specificity, which marks a significant advancement in the field of neuromodulation.

## 3 Discussion

This is the first study to report a non‐magnetostrictive method for magnetoelectric stimulation at the nanoscale that utilizes distinct nanomaterials, BTOs, and MNDs. The use of separate BTO and MND materials, linked via biotin–avidin, simplifies the synthesis process by allowing each component to be independently synthesized and optimized. This avoids the technical challenges of fabricating core–shell or hybrid nanoparticles with lattice‐matched or epitaxially bonded interfaces.^[^ ^12^, ^15^ ^]^ The findings of this study indicate that magnetically driven torques from MNDs induce a piezoelectric response in BTOs, triggering neuronal activity both in vitro and in vivo. Using voltage imaging and fiber photometry, MagTIES is confirmed to effectively trigger neuronal activity within milliseconds and precisely modulate brain oscillations at specific frequencies both in vitro and in vivo. Moreover, the magnetic DBS reported in this study shows higher temporal precision than other state‐of‐the‐art magnetostrictive‐based magnetoelectric DBS technologies reported to date.^[^ ^12^, ^16^, ^17^ ^]^

In addition, the results of this study indicate a size‐dependent effect of MNDs on MagTIES‐induced neuronal responses. Calculations using a simplified model show that larger MNDs can generate a larger torque under an applied magnetic field. The calculated maximum torques that can be generated by MND_250_, MND_220_, and MND_135_ are 5.26 × 10^−18^, 4.1 × 10^−18^, and 1.5 × 10^−18^ Nm, respectively (see Supporting Information). Consistent with these calculations, larger MNDs produce stronger neuronal responses, suggesting that enhancing the MND size further could optimize the MagTIES efficacy. In addition to nanodiscs, nickel–iron permalloy (Ni_80_Fe_20_) magnetic microdiscs (MMDs) can generate a large torque of 188 Oe at 6 Hz for magnetomechanical stimulation in vitro.^[^ ^42^ ^]^ Therefore, using larger nanodiscs or microdiscs might enhance MagTIES‐induced neuronal activity further. These aspects should be considered in future research and applications.

According to the literature, the piezoelectric properties of BTO nanoparticles vary significantly with size. Near‐cubic BTO nanoparticles at the nanoscale (<120 nm) exhibit exceptionally strong piezoelectric responses, whereas larger BTO particles at the bulk scale (300–1000 nm) showed substantially weak piezoelectric properties, approaching those of bulk materials. The experimental findings reported here are consistent with this trend; here, smaller BTOs enabled more robust MagTIES‐induced neuronal responses than larger BTOs. Notably, the stress and strain on the BTOs can be reduced by the damping effect and increased by stress concentration and nonuniform support. Thus, the precise mechanical–electrical transduction mechanisms in MagTIES remain complex. Detailed multi‐physics simulations considering realistic nanoscale interactions are necessary to comprehensively elucidate these effects. Furthermore, alternative mechanisms, such as the movement of intrinsically polarized BTOs relative to the membrane during magnetic field application, cannot be excluded without further analysis. Future studies on refining the MagTIES model should focus on investigating the intrinsic electric polarization dynamics across different BTO sizes.

The spatial positioning of nanomaterials critically influences the effectiveness of MagTIES systems. The proximity of the BTOs to the membrane directly influences the effectiveness of the electric field in modulating the membrane potential. The findings of this study underscore the importance of optimizing the spatial arrangement of BTOs and MNDs to maximize their neuromodulation efficacy. When the BTOs are directly attached to the cell membrane in the BTO_100_/MND_250_ composite, the magnetically induced neuronal responses are significantly stronger (Figure 1d–j) than when the MNDs are placed between the BTOs and cell membrane, highlighting that the BTOs should be in close proximity to the membrane for effective stimulation (Figure 1f–j). Additionally, spatial configuration is critical for precise neuromodulation and ensuring the effective activation of targeted pathways. Although the exact contributions of the magnetomechanical and magnetoelectric mechanisms remain partially understood, the ability of MagTIES to rapidly trigger neuronal responses reinforces its robustness and potential for broad applicability across various systems.

Building on the critical role of spatial arrangement, the detailed mechanism of MagTIES should be investigated further. Unlike core–shell magnetostrictive nanoparticles, the MagTIES system relies on the precise layering of BTO beneath the MNDs. This configuration cannot be pressed into thick pellets of uniform nanoparticles, which is a common approach for increasing the recorded potential during magnetoelectric coefficient measurements,^[^ ^16^, ^17^, ^43^ ^]^ necessitating alternative evaluation strategies. Nevertheless, MagTIES exhibits the most rapid temporal precision among all magnetoelectric stimulation approaches reported to date and operates effectively at the lowest frequency (<100 Hz).^[^ ^16^, ^17^ ^]^ In addition, a strong DC magnetic field is not necessary in the MagTIES system. Low‐frequency (≈10 Hz) operation without DC magnets not only reduces the power requirement of the process but also enhances its safety and biocompatibility, making it suitable for delicate applications in neuromodulation.

The reduced power spectrum intensity observed at 22 and 24 Hz under an AMF at 11 and 12 Hz might reflect the kinetic limitations of the calcium indicator (jGCaMP7s) used in this study. Although jGCaMP7s offer improved brightness and sensitivity compared with earlier GCaMP variants, their relatively long decay time (≈428 ms)^[^ ^44^ ^]^ causes signal overlap at higher frequencies, limiting the resolution of individual calcium transients in the spectral domain. Nevertheless, this indicator is suitable for detecting sustained oscillatory dynamics through power spectrum analysis. Consistent with previous studies, which report that jGCaMP7s can reliably capture rhythmic neural activity at 10 Hz,^[^ ^45^ ^]^ the spectrogram and FFT analyses reported here indicate a robust and statistically significant increase in power near 20 Hz in the BTO/MND group compared with the control group (Figures 5, 6; Figure S12, Supporting Information), confirming that MagTIES can entrain neuronal oscillations in a frequency‐specific manner.

Overall, from in vitro and in vivo studies, this study establishes a proof‐of‐concept for torque‐based magnetoelectric stimulation at the nanoscale using MagTIES, effectively elucidating the detailed mechanism and modulation of in vivo brain oscillations. The results of this study confirm that MagTIES can effectively influence neuronal activity with high temporal precision, offering a potential platform for studying oscillation‐driven mechanisms in neural circuits. Although this study focuses on ≈20 Hz beta‐band modulation, further improvements in nanomaterial properties, magnetic system design, and detection methods may enhance the efficacy of MagTIES further, broadening its potential for investigating neural circuit dynamics, memory, and behavior across a wider range of physiological and pathological conditions.

With regard to biosafety and clinical relevance, both BTO and MNDs are biocompatible materials.^[^ ^46^, ^47^ ^]^ Moreover, in this study, the biosafety of the BTO/MND assemblies was confirmed by measuring cytotoxicity in vitro and immunostaining for microglia and astrocytes in vivo (Figures S6, S9, Supporting Information). As a non‐transgenic approach, MagTIES avoids the complexities and potential risks of gene delivery.^[^ ^48^ ^]^ Future studies should focus on the chronic biocompatibility, long‐term functional stability, and behavioral effects of MagTIES in preclinical models. Exploring the combination of various piezoelectric and superparamagnetic materials can advance the capabilities of MagTIES further. In addition, the scalability and adaptability of the magnetic apparatus used in MagTIES are expected to enable the application of the proposed technique in both fundamental neuroscience research and clinical therapies. In conclusion, MagTIES provides an innovative, less invasive, and precise approach to neuromodulation, particularly in the deep brain regions; its ability to target specific frequencies with high temporal accuracy is expected to open new avenues in neurological research and treatment, setting a foundation for future exploration in this interesting field.

## 4 Experimental Section

### Nanomaterial Synthesis

BTO with different particle sizes (100, 300, and 500 nm) were procured from US Research Nanomaterials. Following the protocol outlined in a previous study, HNDs and MNDs were synthesized using two primary steps. Initially, ethanol (10 mL, 99.5%), anhydrous sodium acetate (0.8 g; Sigma‐Aldrich), and FeCl_3_·6H_2_O (0.273 g; Sigma‐Aldrich) were combined with 0.6, 0.8, and 1.2 mL deionized water (ddH_2_O) to synthesize 250‐, 220‐, and 135‐nm MNDs and HNDs. This mixture was thoroughly stirred to ensure homogenization, sealed in a Teflon‐lined steel vessel, and heated at 180 °C for 18 h in an oven. After heating, the HNDs were sequentially washed twice with ddH_2_O and twice with ethanol and dried in a vacuum desiccator. The synthesized HNDs were either converted into MNDs or set aside for control experiments. In the reduction step, the HNDs (1 mg) were mixed with trioctylamine (20 mL; Acros Organics) and oleic acid (1 g; Sigma‐Aldrich), placed in a three‐neck flask connected to a Schlenk line, and heated to 370 °C for 25 min under a controlled atmosphere of H_2_ (5% with 95% argon; Chiah Lung) and N_2_ (99.9%; Chiah Lung). During this process, a notable color change (from red to dark gray) was observed in the nanodiscs. Once cooled, the discs were washed with hexane (Alfa Aesar), dispersed in chloroform (J.T. Baker), and stored at 4 °C in a glass vial.

### Surface Functionalization and Water Transfer

To functionalize the BTO with an mPEG coating, mPEG‐silane (20 mg) was dissolved in ethanol (9 mL). Subsequently, BTO powder (10 mg) was added and the mixture was sonicated for 2 h. After sonication, the solution was centrifuged in a microcentrifuge at 8500 rpm for 5 min to form pellets. The supernatant was removed and replaced with ddH_2_O. This process, including the sonication of the ddH_2_O refill, was repeated thrice. To functionalize the MNDs and HNDs with a PMAO coating, the nanodiscs, initially in chloroform, were dried in a vacuum desiccator. Next, PMAO (10 mg, 30 000 MW; 419117, Sigma‐Aldrich) was dissolved in chloroform (1 mL), and dried MND or HND powder (1 mg) was added into the mixture followed by sonication for 1 h until the nanodiscs were well suspended in the solution. The PMAO‐coated nanodiscs were dried overnight in a vacuum desiccator. The dried nanodiscs were subsequently added to TAE buffer (Tris‐acetate‐EDTA, Biomate) at a concentration of 25% (w v^−1^) and sonicated at 80 °C for 3 h. After sonication, the particles were pelleted using a microcentrifuge at 8500 rpm for 10 min. The supernatant was removed and the pellet was refilled with ddH_2_O. This sonication‐to‐ddH_2_O refill process was repeated thrice. For neutravidin–BTO surface modification, dried mPEG‐coated BTO (10 mg) was mixed with neutravidin solution (1 mL) for 2 h and centrifuged at 8500 rpm for 3 min; the supernatant was removed followed by the addition of ddH_2_O (3 mL). This process was repeated thrice. Similarly, for biotinylated‐nanodisc surface modification, the dried PMAO‐coated nanodiscs (10 mg) were mixed with biotin buffer (200 µL, 30 µm; Mix‐n‐Stain Reaction Buffer, MXBIOS100, Sigma‐Aldrich) for 2 h and centrifuged at 8500 rpm for 3 min followed by supernatant removal and the addition of ddH_2_O (3 mL). This process was repeated thrice.

### Characterization of Nanomaterials

Initially, the nanodiscs were dried in a vacuum desiccator in chloroform and dissolved in ddH_2_O. For morphological analysis, an aqueous suspension of the BTO or nanodiscs (2 µg mL^−1^) was placed on a copper grid (Ted Pella Inc.) and examined using TEM. An HT7800 (Hitachi) instrument at the Chang Gung Memorial Hospital (CGMH) Microscopy Core Laboratory was used for TEM analysis. The diameter and thickness of the nanodiscs were quantified from TEM images using the Fiji (ImageJ v2.1.0/1.53 c) software. Three diagonals were drawn for each hexagonal nanodisc, and the longest distance was considered to be the diameter measurement.

The crystalline structures of the nanodisc powders were confirmed by XRD using an XtaLAB Synergy DW (Rigaku) machine at the Instrumentation Center of National Tsing Hua University (NTHU). The hematite and magnetite samples were mixed with N‐grease (Apiezon) and positioned on a MicroMeshesTM (MiTeGen) before being analyzed by a HyPix‐Arc 150° curved Hybrid Photon Counting X‐ray detector with graphite‐monochromated Mo Kα radiation (*λ* = 0.71073 Å) at 100 K.

The saturation magnetization of the nanodiscs was studied using a vibrating sample magnetometer to obtain hysteresis curves within a range of ±9 kOe. Magnetic moments were measured using an MPMS SQUID vibrating sample magnetometer (Quantum Design) at the Core Facility Center of National Cheng Kung University (NCKU). An Agilent 725 inductively coupled plasma‐optical emission spectrometer (ICP‐OES) at the NTHU Instrumentation Center was used to quantify the iron concentration for magnetic moment calculations. First, suspensions of BaTiO₃ (2 µL, 20 mg mL^−1^) and Fe₃O₄ (2 µL, 20 mg mL^−1^) were mixed, Subsequently, these materials were separated from the solution using a permanent magnet and washed with ddH₂O (thrice) to remove any residual solution. After magnetic separation, the elemental concentrations, specifically the iron content, of the samples were quantified using ICP‐mass spectrometry (ICP‐MS; THERMO‐ELEMENT XR) at the NCKU Instrumentation Center.

For potential property analysis, PMAO‐coated nanodiscs dissolved in ddH_2_O were examined for zeta potential using electrophoretic light scattering with a DelsaTM Nano C Particle Analyzer (Beckman Coulter). For cellular interaction studies, PMAO‐coated nanodiscs (3.5 µL, 20 mg mL^−1^) were applied to hippocampal neurons for 7 days in vitro (DIVs) for 15 min; this facilitated the attachment of the nanodiscs to the cell membranes. The cells were washed thrice with 1× PBS (Gibco) and fixed using a buffer (3% glutaraldehyde, 2% paraformaldehyde in 0.1 m cacodylate buffer) prepared by CGMH. The fixed samples were stored at 4 °C until analyzed by field emission‐SEM (FE‐SEM) to visualize the attachment of the nanodiscs to neurons. FE‐SEM, conducted on an SU8220 (Hitachi) machine at the CGMH Microscopy Core Laboratory, was used to record nanodisc attachments at 2000× magnification and examined the nanodisc surface features at 30 000× magnification.

### Fluorescence Resonance Energy Transfer Analysis

The linkage of functionalized BTOs and MNDs was analyzed by FRET. To visualize the nanoparticles, neutravidin‐conjugated BTOs were linked to Alexa‐488 by adding neutravidin‐conjugated BTOs (5 mg) to Alexa Fluor 488 dye (200 µL; Thermo Scientific) over 2 h. After centrifugation at 8500 rpm for 5 min, the supernatant was removed and Tyrode's solution (250 µL) was added into the mixture. Biotinylated MNDs were linked to Alexa‐594 by adding biotinylated MNDs (5 mg) to Alexa Fluor 594 dye (200 µL; Thermo Scientific) over 2 h. After centrifugation at 8500 rpm for 5 min, the supernatant was removed and Tyrode's solution (250 µL) was added into the mixture. During the FRET experiment, Alexa‐488‐conjugated BTOs (3 µL, 20 mg mL^−1^) were applied to the primary hippocampal neurons in a 24‐well cultured dish and transferred to a fluorescence microscope (SS‐1000‐00, Scientifica) with W‐view (A12801‐01, Hamamatsu). An LED light source (pE300, CoolLED), Hamamatsu C13440 camera, and filter cubes (DM3000B, Leica) with an AT450/50x excitation filter and AT485DC and AT495lp emission filters were used for imaging. After recording the image of only the BTOs, Alexa‐594‐conjugated MNDs (3 µL, 20 mg mL^−1^) were applied to the cultured neurons in the sample holder of a fluorescence microscope after 10 min. FRET images were recorded using 470‐nm blue light (pE300, CoolLED). FRET images were acquired using a Hamamatsu C13440 camera and W‐view (A12801‐01, Hamamatsu) with an AT450/50x excitation filter and AT485DC and AT495lp emission filters. Using Fiji (ImageJ v2.1.0/1.53c), the FRET ratio was calculated by dividing the red fluorescence intensity by the green fluorescence intensity at a precise location.

### Primary Hippocampal Neuronal Culture

All animal procedures, including neonatal dissection, were approved by the Institutional Animal Care and Use Committee (IACUC) of National Yang Ming Chiao Tung University (NYCU) under protocol number: 108035 (PI: Prof. Po‐Han Chiang). Pregnant Sprague–Dawley rats from LASCO were used in this study. Hippocampal cultures were prepared using pups less than 3 days of age. The hippocampi were extracted in a cold dissection solution consisting of NaCl (160 mm), KCl (5 mm), MgSO_4_ (1 mm), CaCl_2_ (4 mm), HEPES (5 mm), and glucose (5.5 mm) with the pH adjusted to 7.4 using NaOH. Post‐extraction, hippocampus from 2 to 3 pups were combined and placed in a prewarmed digestion solution containing L‐cysteine (1 mM), EDTA (0.5 mm), CaCl_2_ (1 mm), NaOH (1.5 mm), and papain (10 units mL^−1^; 76220, Sigma‐Aldrich) and incubated at 37 °C for 25 min.

The action of papain was halted by removing the digestion solution and incubating the tissues in an inactivation solution composed of bovine albumin (0.25%), trypsin inhibitor (0.25%), D‐glucose (0.4%), and fetal bovine serum (5%; 6140079, Gibco) in Minimum Essential Medium (MEM, w/Earle's salts w/o L‐glutamine; 11090‐081, Gibco) at 37 °C for 2 min. After removing the inactivation solution, the tissues were triturated in a serum medium comprising d‐glucose (0.4%) and fetal bovine serum (5%) in MEM (w/ Earle's salts w/o l‐glutamine) using a fire‐polished glass pipette (111096, Kimble). The dissociated cells were filtered through a cell strainer (93070, SPL) and incubated in a serum medium at 37 °C before seeding. After counting, the cells were seeded onto Matrigel (354234, Corning)‐coated 12 mm coverslips in 24‐well plates at a density of ≈110 000 cells per well. Hippocampal cells were cultured in a neurobasal medium (10888‐022, Gibco) supplemented with B27 (17504‐044, Gibco) and GlutaMAX (35050‐061, Gibco). On the third DIV, a mitotic inhibitor (20 µL, 5‐fluoro‐2’‐deoxyuridine, Sigma; 4 µm in the neurobasal medium) was added to inhibit the growth of glial cells. All imaging and stimulation procedures were conducted between 5 and 14 DIVs.

### Ca^2+^ Imaging and Analysis

Tyrode's solution composed of NaCl (125 mm), KCl (2 mm), MgCl_2_ (2 mm), CaCl_2_ (2 mm), HEPES (25 mm), and d‐glucose (51 mm; Sigma‐Aldrich) was used for all Ca^2+^ imaging experiments on cultured cells. A Fluo‐4 Ca^2+^ Imaging Kit (Invitrogen) was used to measure the Ca^2+^ responses in cultured neurons following the manufacturer's protocol. Neurons were incubated in a Fluo‐4 solution (1 mm) for 15–30 min and then transferred to Tyrode's solution without Fluo‐4 for imaging. For the experiments, functionalized BTO (3 µL, 20 mg mL^−1^) was added to each well containing the solution (497 µL) in a 24‐well plate. Following 5‐min incubation, the cells were washed thrice with Tyrode's solution. Next, functionalized MNDs or HNDs (3 µL, 20 mg mL^−1^) were added into the solution (497 µL) in each well to ensure a final nanodisc concentration of 60 µg per well. After incubation for another 5 min, the cells were washed thrice with Tyrode's solution, and a coverslip with the hippocampal neurons was transferred to a custom stage for analysis under an applied magnetic field in a fluorescence microscope setup (SS‐1000‐00, Scientifica). An LED light source (pE300, CoolLED), Hamamatsu C13440 camera, and GFP filter cube (39002, Chroma) were used for imaging.

For pharmacological experiments, cultured cells were transferred from Tyrode's solution containing Fluo‐4 to an antagonist‐containing Tyrode's solution for imaging. TTX (Abcam) was added to Tyrode's solution at a concentration of 100 nm to block voltage‐gated Na^+^ channels. To concurrently block voltage‐gated Na^+^ and Ca^2+^ channels, a combination of TTX (100 nm) and mibefradil (3 µm, Abcam) was added to Tyrode's solution.

Videos showing Ca^2+^ activity were recorded using an upright fluorescence microscope (SS‐1000‐00, Scientifica) and saved in the “.avi” format using HCImage 4.6.1 (Hamamatsu). The frame rate of the videos was set to 1 Hz. Moreover, the videos were processed using a custom Python script developed on the OpenCV2 platform. A previously described custom Python script based on NumPy, adapting the algorithm from a prior study, was used for converting the fluorescence intensity into Δ*F*/*F*.^[^ ^4^ ^]^ A cell was defined as responding if it exhibited a Δ*F*/*F* greater than three standard deviations from the mean during specified periods. The cell activity rate in each culture sample was determined by calculating the ratio of activated cells to the total number of cells. For detailed information on the custom scripts and methodologies, please refer to the Supporting Information section of a previous publication.^[^ ^4^ ^]^

### Cell Viability Test

Death of primary cultured neurons after multiple magnetomechanical stimulations was measured using PI (P1304MP; Invitrogen). After imaging the multiple MagTIES‐induced Ca^2+^ responses of primary cultured neurons with Fluo‐4, PI was added to the extracellular solution to ensure a final concentration at 120 µm. After 5 min, the fluorescence of Fluo‐4 and PI were imaged using a fluorescence microscope (SS‐1000‐00; Scientific). An LED light source (pE300, CoolLED), a Hamamatsu C13440 camera, and filter cubes (39002 and 39010, Chroma) were used for imaging. For live/dead cell assessment in HEK293T cells, a Calcein‐AM/PI assay was performed. Calcein AM (C3100MP, Invitrogen) was added to the extracellular solution at a final concentration of 2 µm following repeated MagTIES stimulation and calcium imaging. Cells were incubated at 37 °C for 15–30 min to allow intracellular esterases to convert Calcein AM into fluorescent calcein. The resulting fluorescence signal was imaged under the same microscope setup described above.

To investigate the endocytosis of BTO and MND in the cells, neurons were treated with BTO (60 µg) and MND on days 0, 3, and 7. After each treatment, the cells were washed thrice with PBS to remove excess material. The cells were processed for TEM after washing. The subsequent procedures involved fixing the cells with glutaraldehyde (3%) and paraformaldehyde (2%) in cacodylate buffer (0.1 m, pH 7.4) at 4 °C followed by rinsing with cacodylate buffer (0.1 m) for 10 min; this process was repeated thrice at 4 °C. Cells were then post‐fixed with osmium tetroxide (OsO₄) (1%) in cacodylate buffer (0.1 m) for 1 h at 4 °C, rinsed again with the same buffer, and stained with uranyl acetate (4%) for 2 h at room temperature (20 to 26 °C) while protecting them from light. The samples were further processed by dehydration and embedding prior to TEM imaging.

### Voltage Imaging and Analysis

In this study, di‐8‐ANEPPS (2 mM, Invitrogen) was used as a voltage‐sensitive dye for the photometric measurements of the real‐time changes in membrane potential. Before voltage imaging, di‐8‐ANEPPS (1 µL) was directly added to primary hippocampal neurons in a 24‐well culture dish and incubated for 30 min. Next, functionalized BTO (3 µL, 20 mg mL^−1^) was added to each well over 5 min followed by washing thrice with Tyrode's solution. Subsequently, functionalized MNDs (3 µL, 20 mg mL^−1^) were added to each well to ensure a final nanodisc concentration of 60 µg per well. After another 5 min of incubation in a CO_2_ incubator, the cells were washed thrice with Tyrode's solution. Subsequently, the coverslip with the hippocampal neurons was transferred to a custom stage for analysis under an applied magnetic field in a fluorescence microscope setup (SS‐1000‐00, Scientifica) equipped with a W‐view (A12801‐01, Hamamatsu), an LED light source (pE300, CoolLED), a Hamamatsu C13440 camera, Leica filter cubes (DM3000B) with an AT450/50× excitation filter, and AT485DC and AT495lp emission filters for imaging. The images were divided into 2048 × 32 pixels, and 470 nm blue light was used for voltage imaging at ≈1000 frames per second. Fiji (ImageJ v2.1.0/1.53c) was used to select the region of interest (ROI) on the cell membrane and measure the fluorescence intensity. The green‐to‐red fluorescence intensity ratio was used to monitor voltage changes in the membrane potential.

### Magnetic Apparatus for Fluorescence Microscope

An air–core magnetic coil with 4‐cm height, 13.5‐cm outer diameter, and 3.5‐cm inner diameter was constructed using 2000 turns of an 18 AWG self‐bonding copper wire (SBWR, Chientai). This coil showed a resistance of 7 Ω and an inductance of 11 mH. A current of 2.7 A was used for 10 Hz, 50 mT magnetic stimulation. A custom‐made H‐bridge driver was used to generate different magnetic fields using this coil. This H‐bridge comprised two p‐channel MOSFETs (IRF4905, International Rectifier) and two n‐channel MOSFETs (IRF3710, International Rectifier). Two additional n‐channel MOSFETs were implemented to control the p‐channel MOSFETs within the H‐bridge. The operational voltage range of the circuit could be adjusted using two pairs of resistances and MOSFET parameters, allowing easy modification by replacing different resistances or MOSFETs. A function generator (33210A, Keysight) produced ±5 V square waves. Signals from the function generator were passed through a voltage follower and an inverter (TLC2272, Texas Instruments) with gains equal to one. The voltage follower and inverter outputs were 180° out of phase, with each signal controlling half of the full‐bridge driver. An internal power supply (PS‐3030DF model, LONGWEI) was used to power the op‐amps on the custom‐made H‐bridge driver. In contrast, an external power supply (IT6721, ITECH) was used to generate magnetic fields in the coil.

To evaluate the magnetic fields generated by the coils, the Finite Element Method Magnetics software (version 4.2) was used to simulate the magnetic fields of the coil in 2D. The magnetic problem mode and planar configuration in FEMM4.2 were used in the program setting. The parameters of the copper wire (12 or 18 AWG) and air were obtained from the material library in FEMM4.2. A Gaussian meter (TM801, KANETEC) was used to measure the magnetic field in the coil, and an axial probe was used to detect the magnetic field generated inside the coil.

### Animal Surgery

All animal procedures were approved by the Institutional Animal Care and Use Committee (IACUC) of National Yang Ming Chiao Tung University (NYCU) under protocol number: 108035 (PI: Prof. Po‐Han Chiang). All experiments were conducted in accordance with the Guide for the Care and Use of Laboratory Animals of NYCU. All mice were purchased from BioLASCO Taiwan Co. Ltd. and maintained under a 12‐h light–dark cycle at the NYCU Laboratory Animal Center before the experiments. Moreover, 8 to 12‐week‐old C57BL/6 male and female mice were used in all in vivo experiments. Mice were anesthetized with 1.5–2% isoflurane in oxygen and placed on a stereotaxic frame. In addition, an ophthalmic ointment was applied to prevent the eyes from drying, and the body temperature was maintained using a regulated heated pad.

For the c‐fos experiment, neutravidin‐conjugated BTOs and biotinylated nanodiscs (MNDs or HNDs) were sequentially and unilaterally injected into the lateral basolateral amygdala (AP −1.75 mm, DV −4.80, and −4.7 mm; ML −3.15 mm from bregma). A microinjection syringe (7653‐01, Hamilton) with a 33GA needle (7803‐05, Hamilton) and a micropump (LEGATO130, Kd Scientific) with injection rates of 100 nL per min were used to inject each point with different concentrations of BTOs, MNDs, or HNDs (1 µL each) in PBS. After cranial surgery, Carprofen (0.2 mL, PHR1452, Sigma‐Aldrich) was administered to mice through subcutaneous injection to relieve post‐surgical pain. MagTIES stimulation was conducted one day after surgery.

For photometry experiments, the virus was injected by a microinjection syringe (7653‐01, Hamilton) with a 33GA needle (7803‐05, Hamilton) and a micropump (LEGATO130, Kd Scientific) at 100 nL per min with the following coordinates: the virus was injected unilaterally into the basolateral amygdala (AP −1.55 mm, DV −4.75 mm; ML +3.15 mm from bregma) A total viral volume of 500 nL was delivered to the site; the needle was kept in the site for at least 15 min to permit diffusion. Following injection, the needles were progressively removed. After one month of viral expression, Neutravidin‐conjugated BTOs and biotinylated nanodiscs (MNDs or HNDs) were sequentially and unilaterally injected into the basolateral amygdala (AP −1.55 mm, DV −4.75 mm; ML +3.15 mm from bregma). The BTOs and nanodiscs (MNDs or HNDs) were injected at rates of 80 and 60 nL per min, respectively. On the same day, optic fibers (Inper LLC) with 400‐µm diameter, 5‐mm length, and 0.5 NA were implanted into the basolateral amygdala (AP −1.55 mm, DV −4.5 mm; ML +3.17 mm from bregma) and secured with dental cement (Superbond, Sun Medical). The mice were then kept warm using a heating pad, and the analgesic carprofen was administered through subcutaneous injection post‐surgery. Photometry was conducted after a one‐week recovery period. All photometry and MagTIES experiments were conducted 1–3 weeks after surgery.

### Fixed Brain Slicing and Immunostaining

To observe c‐fos expression after stimulation, the nanodiscs injected into C57BL/6 male and female mice were placed in a custom‐made coil for magnetic stimulation. The mice were sacrificed 90 min after magnetic stimulation. All brains were collected after transcranial perfusion with Perfluoroalkoxy alkanes (PFA) (4%) in PBS. Coronal brain sections were sliced into 60‐µm sections by a vibratome (5100 MZ, Campden) with an amplitude of 0.5 and frequency of 50 Hz. The brain slices were washed thrice with PBS for 5 min and permeabilized with 2% (v/v) Triton X‐100 (Sigma‐Aldrich) for 20 min. The background was cleared using Triton‐X‐100 (2%), H_2_O_2_ (30%), and MeOH for 10 min. After PBS washing, the slices were blocked with 3% normal goat serum in PBS for 60 min at room temperature (20 to 26 °C). The slices were washed thrice with PBS. For immunostaining, slices were incubated with the first antibody solution containing 1:750 rabbit anti‐c‐fos monoclonal antibody (9F6#2250, Cell Signaling), normal goat serum (1%), and Triton‐X 100 (2%) in PBS. Slices were incubated at 4 °C for 16–18 h. After three washes with PBS, the slices were incubated with appropriate secondary antibodies in PBS (1:500 goat anti‐rabbit Alexa Fluor 594 (ab150080, Abcam)). After washing thrice with PBS, the slices were mounted on glass microscope slides using a mounting medium containing 4',6‐diamidino‐2‐phenylindole (DAPI) (GTX30920, Genetex). An upright fluorescence microscope (SS‐1000‐00, Scientifica), LED light source (pE300, CoolLED), Hamamatsu C13440 camera, and filter cubes (39000, 19008, 31002, Choursoma) were used for imaging.

For Iba1, CD68, and GFAP staining in the amygdala, 50 µm brain slices were placed in a 24‐well plate. After washing thrice with PBS (for 5 min each), the slices were permeabilized with 2% (v/v) Triton X‐100 (Sigma‐Aldrich) for 30 min. After applying a mixture of Triton‐X‐100 (2%), H_2_O_2_ (30%), and methanol onto the slices for 10 min to remove tissue impurities, the slices were washed thrice with PBS, blocked with normal goat serum (3%) in PBS for 60 min at room temperature (20 to 26 °C), and washed thrice with PBS. Depending on the group, the slices were then incubated with primary antibodies: rabbit anti‐Iba1 antibody (ab153696, Abcam) at a 1:1000 ratio; rat anti‐CD68 antibody (AB53444, Abcam) at a 1:750 ratio; or rabbit anti‐GFAP antibody (E4L7M#80788, Cell Signaling) at a 1:1500 ratio. These antibodies were prepared in a solution of normal goat serum (1%) and Triton‐X 100 (2%) in PBS. The slices were incubated at 4 °C for 16–18 h, washed thrice with PBS, and incubated with secondary antibodies for 2 h. The following secondary antibodies were used: goat anti‐rabbit Alexa Fluor 594 (ab150080; Abcam) at a 1:500 ratio and goat anti‐rat Alexa Fluor 594 (ab150160; Abcam) at a 1:500 ratio. All slices were washed thrice with PBS before mounting on glass microscope slides using a mounting medium containing DAPI (F6057, Sigma). An upright fluorescence microscope (SS‐1000‐00, Scientifica) equipped with an LED light source (pE300, CoolLED), Hamamatsu C13440 camera, and filter cubes (39000, 19008, 31002, Chroma) was used for recording images.

The immunofluorescence images of Iba1, CD68, and GFAP were processed and analyzed using a systematic approach. First, the folder path and storage location of the image files were specified to load the images for analysis. TIFF‐formatted files in the specified directory were sequentially imported. Statistical data, such as the area, mean intensity, minimum intensity, maximum intensity, and standard deviation, were calculated and recorded for each image. The pixel intensity values were adjusted by subtracting 10 from the minimum and adding 10 to the maximum values, ensuring that the adjusted intensity values remained within the range of 0–65535. Following this adjustment, the stained areas in the images were analyzed using the “triangle” automatic threshold setting method in ImageJ, setting the size range of the stained areas within 0 to infinity. The results were standardized and displayed. Each processed image was saved in the output directory using a specific filename. After processing, each image was closed and the script automated the entire workflow. The final results were presented as follows: “Image Name, Area, Mean, Min, Max, Standard Deviation.”

### Fiber Photometry Measurement of Fluorescence in Freely Moving Mice

After virus injection and fiber‐optic implantation, the mice were returned to their home cages for more than seven days. A fiber photometry system (R810, RWD Life Science Co. Ltd. China) was used to record the green fluorescence signals (GCaMP and isosbestic wavelengths) at excitation wavelengths of 470‐ and 410‐nm from the LED. Calcium fluorescence signals were acquired at 300 Hz using alternating pulses from both light sources. The recorded parameter settings indicate that the 410 nm light was set at 38.11%, while the 470 nm light was configured at 17.18% to ensure the least amount of photobleaching while allowing sufficient detection of the calcium response. A digital camera was used for behavioral recordings that were synchronized with the calcium signal recordings. Experiments were conducted using the above‐mentioned parameters and equipment to record the fluorescence signals of the neurons in the target region. To minimize environmental stress, the mice were allowed to acclimate to their surroundings in a behavioral laboratory for >1 h. Prior to recording fiber photometry measurements, the mice were fitted with sleeves and monochannel optical fibers and returned to their cages for 15 min for habituation. Subsequently, they were placed inside the experimental cylinder within the 10‐cm coil for an additional 15 min to acclimate to the stimulation environment, thereby mitigating the impact of environmental influences. After this habituation period, the baseline fluorescence was recorded for 4 min without the application of magnetic fields. Moreover, the fluorescence was recorded twice under magnetic stimulation. The stimulation conditions and durations were adjusted according to the experimental group.

### Fast Fourier Transform (FFT) and Spectrogram Analysis

In this study, the time‐dependent frequency components of recorded signals were analyzed using a combination of high‐pass filtering and spectrogram visualization. The signal data were acquired from CSV files representing fluorescence measurements sampled at a frequency determined from the timestamp data. The raw signals were preprocessed to isolate the relevant frequency components using a fifth‐order Butterworth high‐pass filter with a cutoff frequency of 4 Hz. This filtering step removed low‐frequency noise while preserving high‐frequency oscillations, which were critical for analysis. Filter design and implementation were conducted using SciPy in Python 3.12. After filtering, a spectrogram was generated to visualize the frequency content of the signal over time. The spectrogram, computed using FFT, provided a time‐frequency representation of the signal, enabling the identification of dominant frequencies and their evolution throughout the recording. A frame size equal to the sampling frequency was used for analysis, and the power spectral density was computed and visualized on a linear scale. The spectrogram was plotted in the frequency range of 4–40 Hz and represented using a jet‐color map. The resulting heat maps showed the power distribution across different frequencies over time, with higher‐power regions indicating the presence of significant frequency components. All data processing and visualization were conducted using the Python libraries numpy, scipy, and matplotlib. This workflow allowed the efficient processing of large datasets and noise filtration, enabling a detailed analysis of the dynamic frequency behavior of the signals.

### Magnetic Apparatus for In Vivo Experiments

A custom‐made magnetic apparatus with two air–core coils was used for c‐fos experiments to generate a uniform magnetic field for wireless neuronal stimulation in vivo. A custom‐made magnetic apparatus with four air–core coils was used for fiber photometry experiments. The height, outer diameter, and inner diameter of each air–core coil were 4, 21, and 11 cm, respectively. On stacking, the gap between the coils was 1 cm, and each coil comprised 350 turns of 12 AWG copper AWGs. The resistance and inductance of each coil were within 1.02–1.55 Ω and 22–31.6 mH, respectively. Full bridge modules (AQMH3615NS, AKELC) were used as drivers for each coil. The drivers were controlled by 5‐V square waves generated by an Arduino UNO (Arduino). A custom‐made script with an Arduino IDE controlled the Arduino board. To provide the working voltage for the complete bridge module setup, an internal power supply of 5 V was provided by an Arduino. In addition, an external power supply (HJS‐1000, Huntkey) was used to supply the coil currents. The magnetic field intensity in the coils was measured using a Gaussian meter (TM801, KANETEC) before the animal experiments.

### Statistics Analysis

In this study, the standard error of the mean (s.e.m.) was used to ensure an accurate estimation of population means from sample data. The s.e.m. provided a clear measure of precision,^[^ ^49^ ^]^ allowing the drawing of reliable inferences and robust comparisons between group means. The JASP software (v0.15.0.0, v0.18.0.0, JASP team) was used for all statistical analyses. All error bars in the dot plots and all gray and light areas in the fluorescence changes and power intensity traces indicate s.e.m. values. The paired and unpaired *t‐*tests were used to compare paired and independent data, respectively. Tukey's post‐hoc test and one‐way ANOVA were used to compare independent data from multiple groups.

## Conflict of Interest

P.C. and C.C. have filed patents in Taiwan (I842610) and the U.S. (application No. 18/540750) describing the magnetic field‐induced electrical stimulation of cells, which was related to this research.

## Author Contributions

Formal analyses was done by P.C., C.C., G.T., and L.C. Funding acquisition was done by P.C. Investigation was done by P.C., C.C., G.T., L.C., and Y.T. Methodology was done by P.C., C.C., G.T., L.C., and J.H. Project administration was done by P.C. Supervision was done by P.C. Validation was done by P.C., C.C., G.T., and L.C. Visualization was done by P.C., C.C., G.T., L.C., and J.H. Writing the original draft was done by P.C. P.C., C.C., and G.T. wrote the review, and editing.

## Supporting information



Supporting Information

## Data Availability

The data that support the findings of this study are available from the corresponding author upon reasonable request.
