# Supplementary material for: Magnetic‐Driven Torque‐Induced Electrical Stimulation for Millisecond‐Scale Wireless Neuromodulation
Source: Adv Healthc Mater. 2025 Jun 16;14(20):2500805. doi: 10.1002/adhm.202500805 (PMC12333473; doi:10.1002/adhm.202500805)
Supplement: Supplementary file 1 — Supporting Information [file ADHM-14-0-s001.docx]

Supporting Information for

**Magnetic-driven Torque Induced Electrical Stimulation for Millisecond-Scale Wireless Neuromodulation**

Chao-Chun Cheng^1^, Li-Ling Chen^1^, Guan-Jhong Tseng^1^, Jun-Xuan Huang^1^, Yen-Jing Ting^1^, Po-Han Chiang^1^*

^1^Institute of Biomedical Engineering, National Yang Ming Chiao Tung University, Taiwan (R.O.C.)

*corresponding author, Email: [phc@nycu.edu.tw](mailto:phc@nycu.edu.tw)

**The PDF file includes:**

Supporting Methods

Supporting Figures. S1 to S12

**Supporting Methods**

**Calculation of Magnetic Field-induced Torque and Force on MNDs**

Based on the analysis of magnetically induced forces from MNDs during alternating magnetic field exposure in a previous study^[3]^, torque generation is considered the dominant mechanical effect, compared to other hypotheses such as inter-particle attractive forces and magnetic gradient forces. Following a simplified model from that study, we calculated the torque exerted by MNDs of different sizes.

Here, we assume full in-plane magnetization of the MNDs and an optimal orientation with a 90° angle between the magnetic dipole moment and the external magnetic field. This torque is critical for understanding the mechanical-to-electrical energy conversion process in BTO nanoparticles.

The volume (V) of an MND_250_ was calculated based on its geometry, with a radius (r) of 125 nm and a height (h) of 32 nm, as follows:

|  | $V\approx\pi r^{2}h$ | (1) |
| --- | --- | --- |
|  | $V\approx\pi\times\left( 125{\times10}^{-9}m \right)^{2}\times\left( 32{\times10}^{-9}m \right)=1.57{\times10}^{-21}m^{3}$ | (2) |
|  |  |  |

Given the volume, the magnetic moment $\mu$ is derived using the density (ρ) and magnetization (M) of the MNDs.

|  | $\left\vert\mu\right\vert=V\rho M$ | (3) |
| --- | --- | --- |

The density of magnetite is 5150 kg·m^-^³. The magnetization (M ≈ 13 A·m²·kg^-1^ at 50 mT) was obtained from vibrating sample magnetometry (VSM) measurements (Fig. S1d). The magnetic moment ($\mu$) was calculated by:

|  | $\left\vert\mu\right\vert=\left( 1.57{\times10}^{-21}m^{3} \right)\times5150\frac{\mathrm{kg}}{m^{3}}\times13\frac{Am^{2}}{\mathrm{kg}}=1.05{\times10}^{-16}Am^{2}$ | (4) |
| --- | --- | --- |

The torque (τ) exerted on the MNDs in a uniform magnetic field ($B$) is then calculated:

|  | $\tau=\mu\times B$ | (5) |
| --- | --- | --- |

When there is an angle between the magnetic dipole ($\mu)$and field ($B)$, the torque is:

|  | $\vert\tau\vert=\vert\mu\vert\vert B\vert\sin\theta_{\mu-B}$ | (6) |
| --- | --- | --- |

When the angle between magnetic moment and magnetic field is 90˚, it has the maximum torque. In our study, when apply 50 mT to a uniformly magnetized MNDs, the maximum torque is:

|  | $\left\vert\tau\right\vert=1.05{\times10}^{-16} Am^{2}\times0.05 T\times\sin90˚=5.26{\times10}^{-18}Nm$ | (7) |
| --- | --- | --- |

With the maximum torque at 5.26×10^-18^ Nm. The force can be derived from torque:

|  | $F=\frac{\vert\tau\vert}{r}$ | (8) |
| --- | --- | --- |

The force on the edge of MNDs with 125 nm radius is:

|  | $F=\frac{\vert\tau\vert}{r}=\frac{5.26{\times10}^{-18}Nm}{125{\times10}^{-9}m}= 4.2{\times10}^{-11}N$ | (9) |
| --- | --- | --- |

From the calculation, the maximum torque generated by MND_250_ with 50 mT is 5.26×10^-18^ Nm. The force on the edge of MND_250_ is 4.2×10^-11^ N. With the same model, the maximum torque generated by MND_220_ and MND_135_ are 4.1×10^-18^ Nm and 1.5×10^-18^ Nm, respectively. The corresponding edge forces for MND_220_ and MND_135_ are 3.7×10^-11^ N and 2.3×10^-11^ N, respectively.

However, unlike simple core-shell magnetoelectric nanomaterials, accurately calculating the strain and stress generated on the BTO by the torque from the MND is challenging. This complexity arises from factors such as mechanical damping, stress concentration at the nanoscale interface, and potential non-uniform support conditions. Further experimental and computational studies will be necessary to fully elucidate the mechanical-to-electrical transduction mechanisms underlying the MagTIES system.

**Supporting Figures**


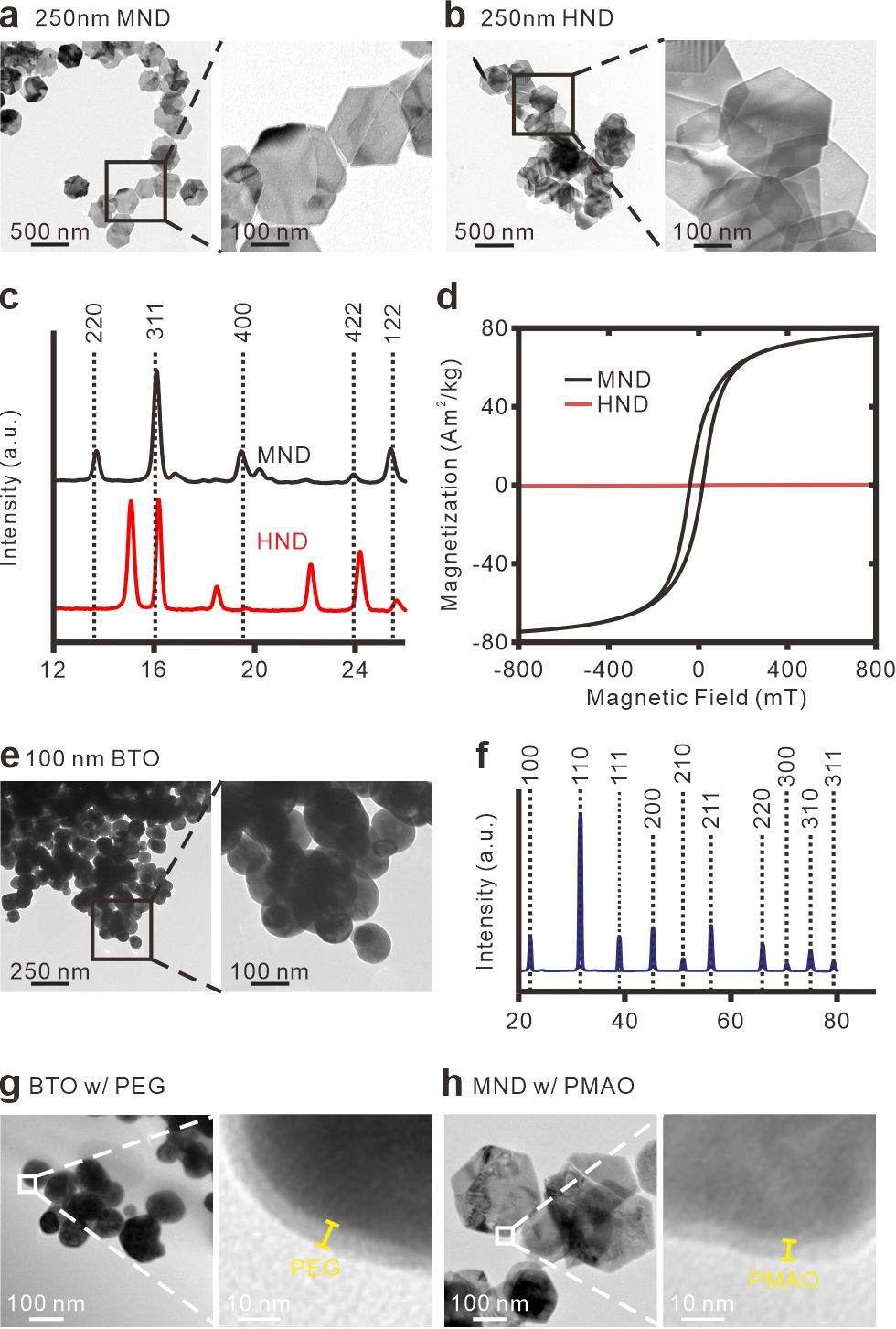


**Figure S1. Characterization of the properties of nanomaterials. a,** TEM image of MND_250_. **b,** TEM image of HND_250_. **c**, XRD traces of MNDs and HNDs. **d**, VSM results of MNDs and HNDs. **e,** TEM image of BTO_100_. **f**, XRD traces of BTOs. **g,** TEM image of the coating layer of PEGylated BTO_100_. **h,** TEM image of the coating layer of PMAO-coated MND_250_.


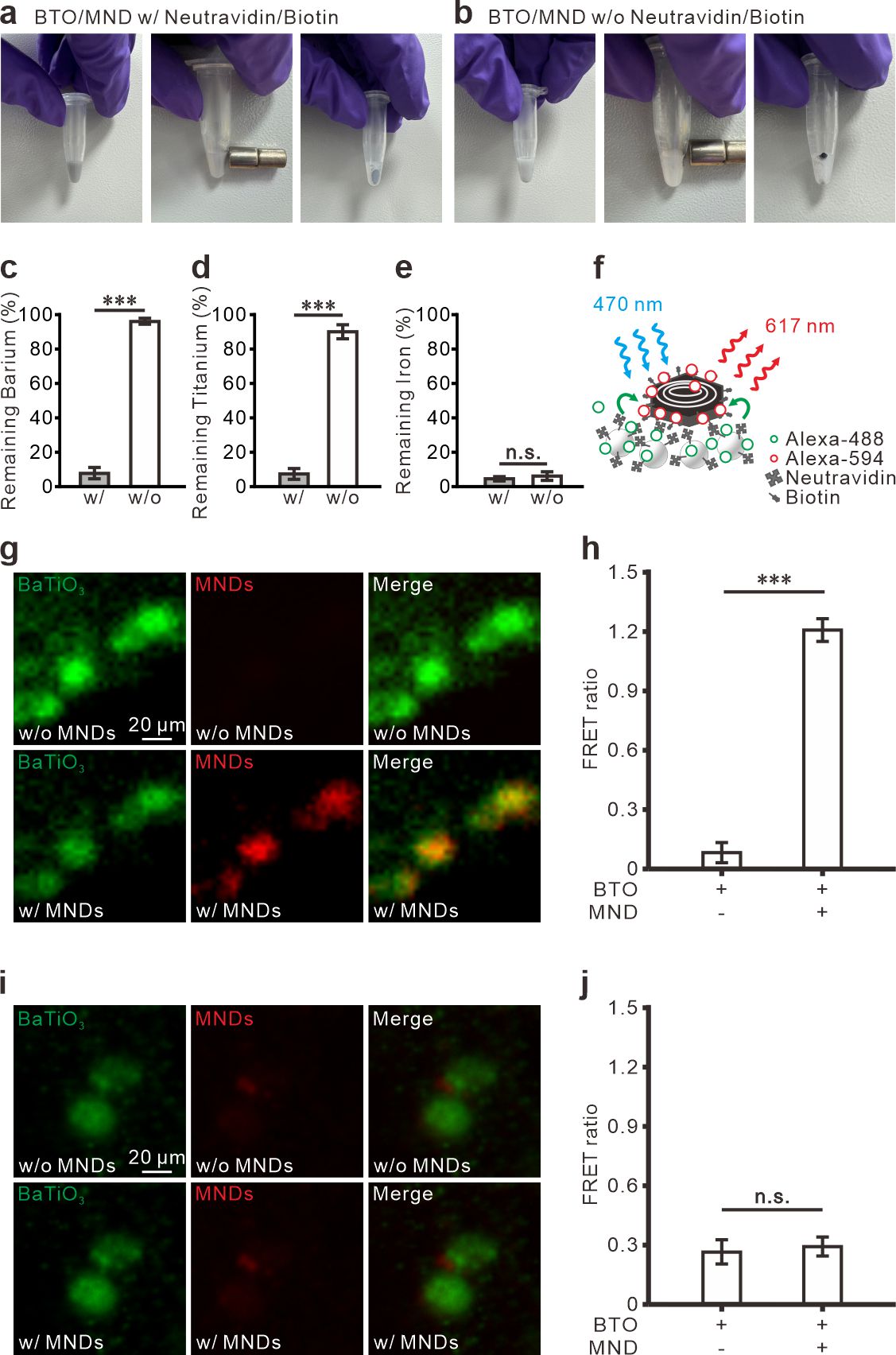


**Figure S2. Characterization of the linkage of nanomaterials. a,** Removing BTO/MNDs in the mixture of BTO/MND with neutravidin/biotin by a permanent magnet. Left, BTO/MND mixture before using magnet. Center, using a magnet to attract BTO/MND. Right, a gray pellet was attached to the wall when removing the magnet. **b,** Removing MNDs in the mixture of BTO/MND without neutravidin/biotin by a permanent magnet. Left, BTO/MND mixture before using magnet. Center, using a magnet to attract MNDs. Right, a black pellet was attached on the wall then removing the magnet. **c-e,** Elements in the remaining solution were divided by the total amount of elements in the pellet and in the remaining solution, n = 3 in all groups. ***p < 0.001, unpaired t-test. **f,** Schematic of FRET. Alexa-488-conjugated BTOs and Alexa-594-conjugated MNDs were binding by biotin-avidin linkage. When 470 nm blue light was applied, the green fluorescence from Alexa-488 was transferred to Alexa-594, emitting red fluorescence. **g,** FRET image of neurons with fluorophore-conjugated BTO_100_/MND_250_ with neutravidin/biotin. Top, Neurons with Alexa-488-conjugated BTOs before application of Alexa-594-conjugated MNDs. Bottom, Neurons with Alexa-488-conjugated BTOs and Alexa-594-conjugated MNDs. Left, Green fluorescence excited by 470 nm blue light. Middle, Red fluorescence excited by 470 nm blue light. Right, Merged image. **h,** FRET ratio (red intensity/ green intensity) of Alexa-488-conjugated BTOs treated neurons with and without Alexa-594-conjugated MNDs. n = 10, sample = 3 in both groups. ***p < 0.001, paired t-test. **i,** FRET image of neurons with fluorophore-conjugated BTO_100_/MND_250_ without neutravidin/biotin. Top, Neurons with Alexa-488-conjugated BTOs before application of Alexa-594-conjugated MNDs. Bottom, Neurons with Alexa-488-conjugated BTOs and Alexa-594-conjugated MNDs. Left, Green fluorescence excited by 470 nm blue light. Middle, Red fluorescence excited by 470 nm blue light. Right, Merged image. **j,** FRET ratio (red intensity/ green intensity) of Alexa-488-conjugated BTOs treated neurons with and without Alexa-594-conjugated MNDs. n = 10, sample = 3, in both groups.


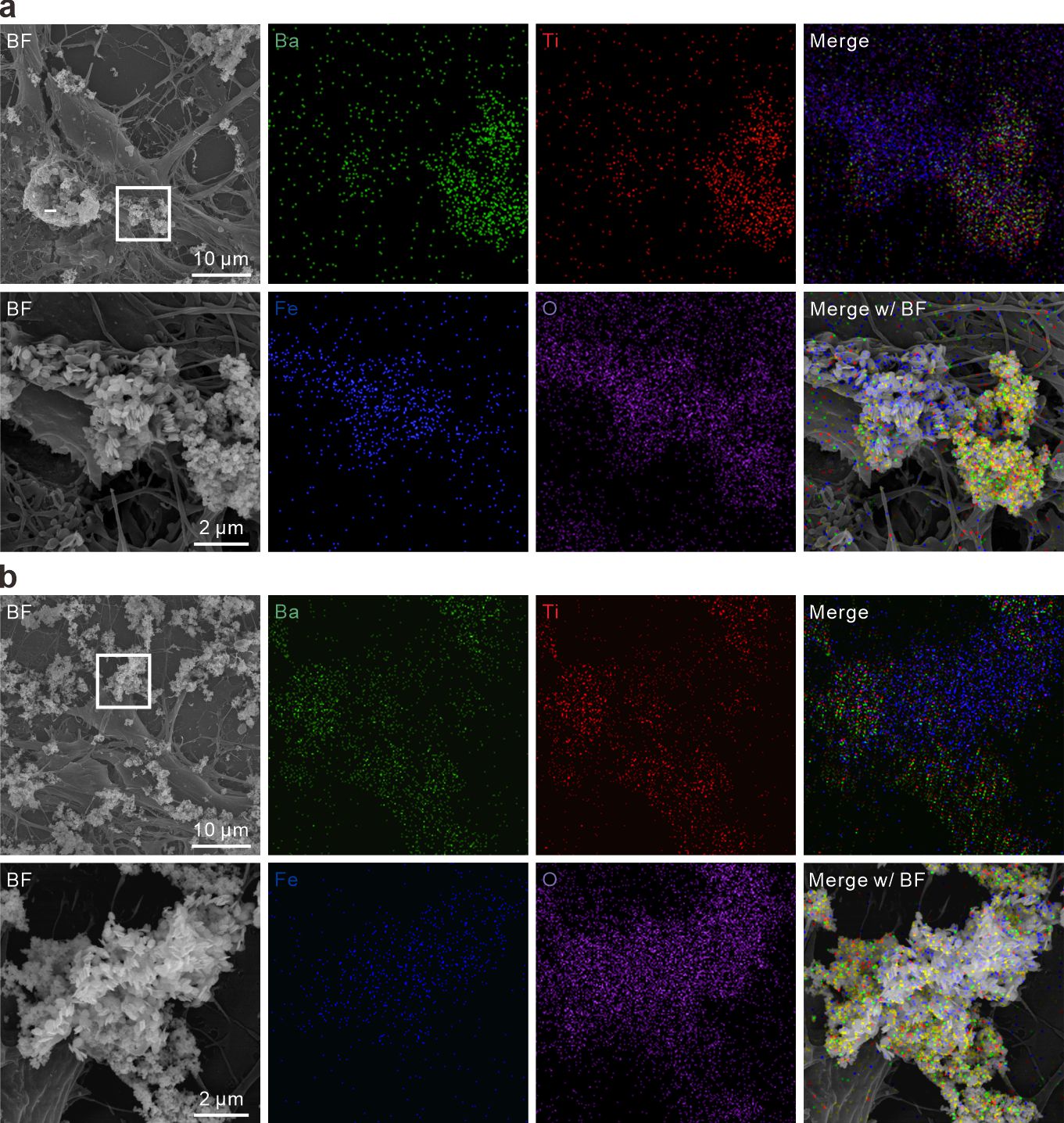


**Figure S3. EDS-SEM image of nanomaterials on the neurons. a,** Bright-field (BF) scanning electron microscopy (SEM) images (left) of BTO/MND attaching to neurons. Elemental distribution maps obtained from energy dispersive spectroscopy (EDS) are shown for Barium (Ba, green), Titanium (Ti, red), Iron (Fe, blue), and Oxygen (O, purple). The merged images (right) display the spatial colocalization of these elements. The lower panel includes a higher magnification view of the boxed region in the upper BF image, with a merge of the elemental maps superimposed on the BF image. **b,** Bright-field (BF) scanning electron microscopy (SEM) images (left) of BTO/HND attaching to neurons. Elemental distribution maps obtained from energy dispersive spectroscopy (EDS) are shown for Barium (Ba, green), Titanium (Ti, red), Iron (Fe, blue), and Oxygen (O, purple). The merged EDS maps illustrate the distribution of Ba, Ti, Fe, and O across the nanomaterials and their localization on neurons.


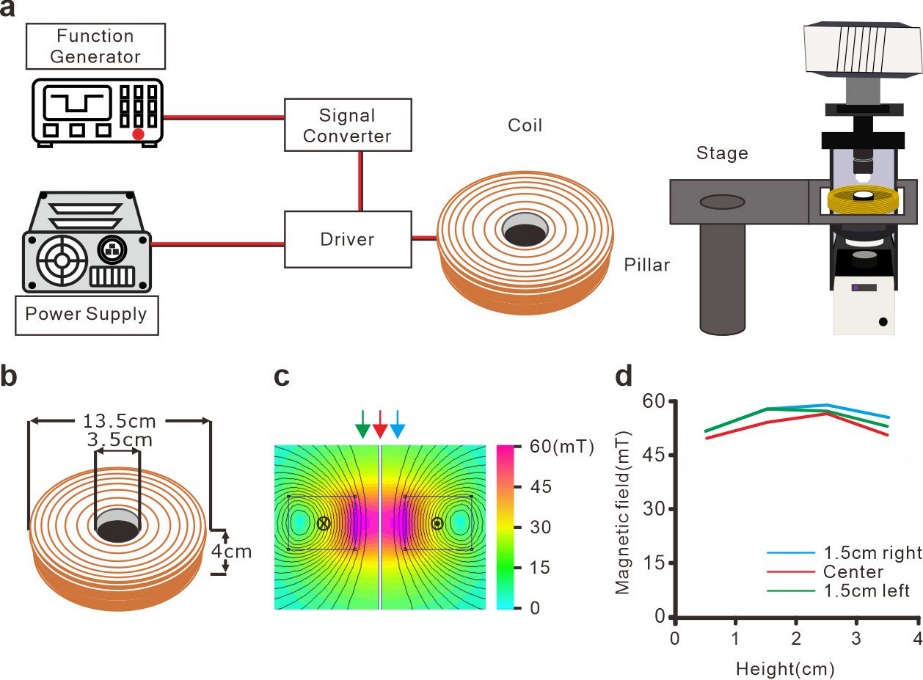


**Figure S4. Magnetic apparatus for upright fluorescence microscope. a,** Scheme of magnetic setup. **b,** Dimension of magnetic coil for fluorescence microscope. **c,** Heatmap of FEMM simulation for magnetic field distribution. **d,** Measured magnetic intensity from the coil by Gaussmeter. The measurement was vertically along the arrow indicated location in (**c**).

**
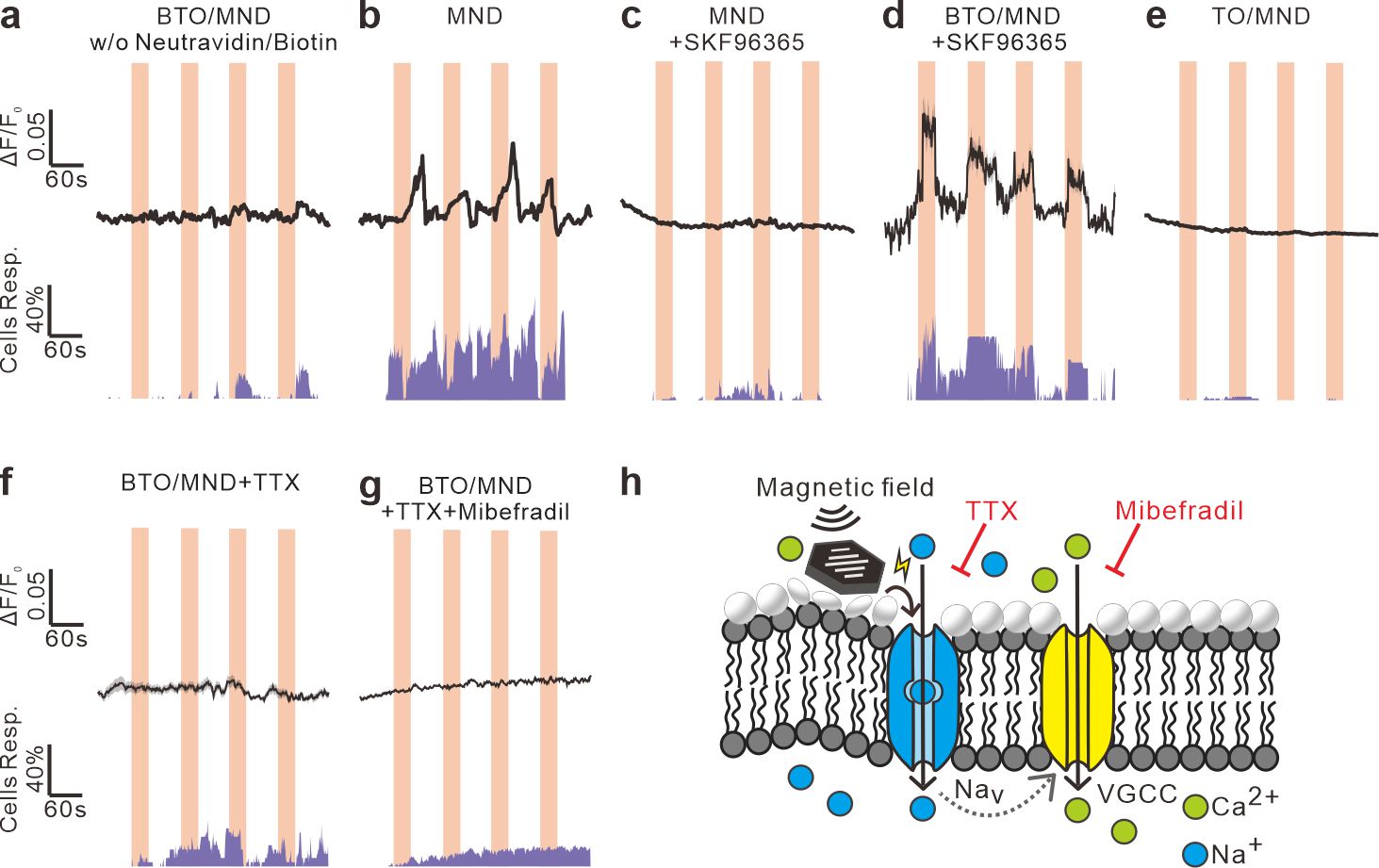
**

**Figure S5. MagTIES responses in cultured neurons with different condition. a** to **g,** Fluorescence change (top) and cell response rate (bottom) of MagTIES in cultured neurons with BTO/MND w/o neutravidin/biotin (a, n = 70, sample = 6), MND alone (b, n = 60, sample = 6), MND + SKF96365 (c, n = 123, sample = 8), BTO/MND + SKF96365 (d, n = 51, sample = 6), TO/MND (e, n = 71, sample = 6), BTO/MND + TTX (f, n = 30, sample = 6), and BTO/MND + TTX + Mibefradil (g, n = 38, sample = 6). Light orange area, periods of 50 mT AMF at 10Hz. **h,** Schematic of hypothesis. The MagTIES can trigger the activation of the voltage-gated Na^+^ channel or voltage-gated Ca^2+^ channel on the membrane.


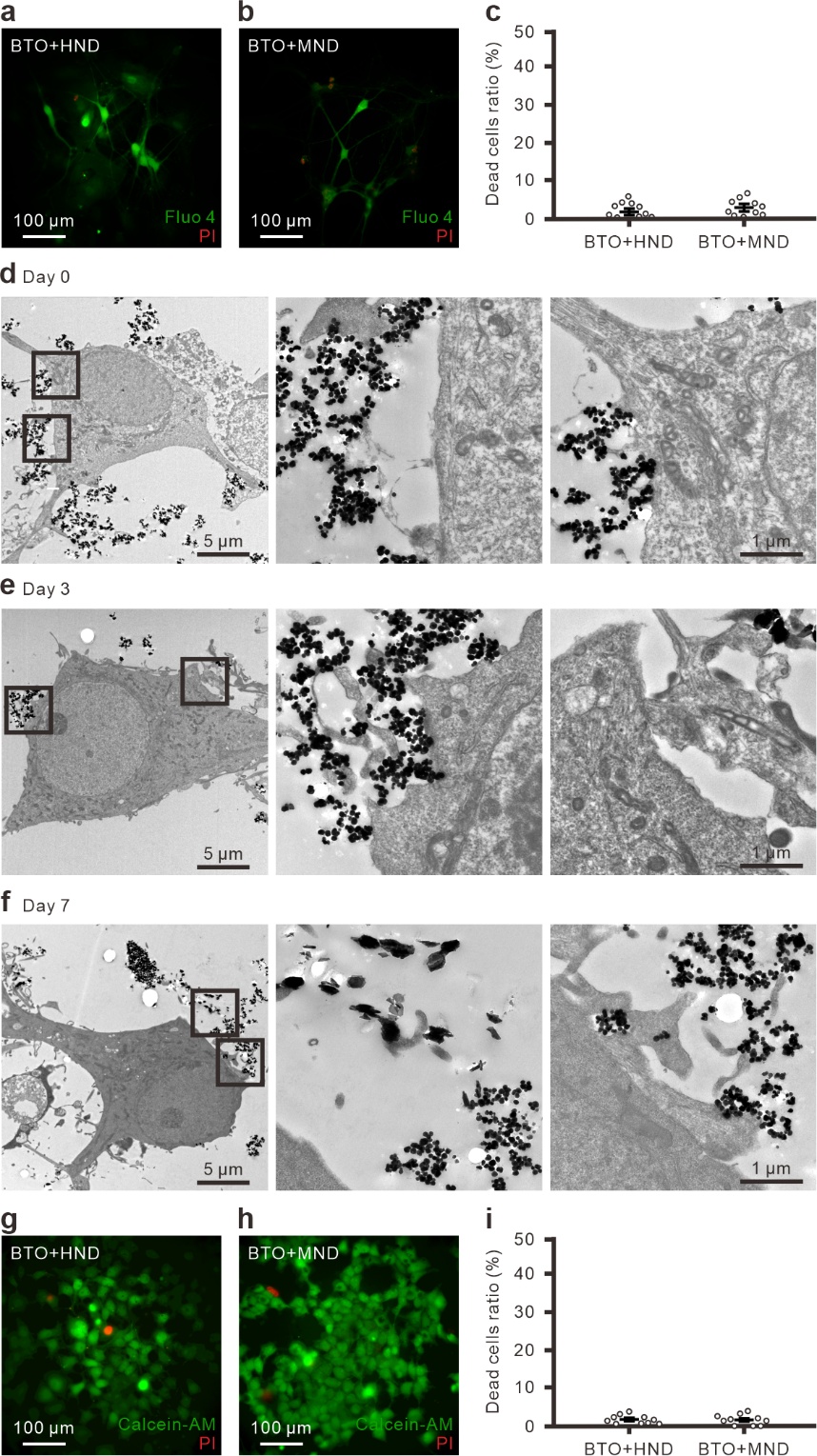


**Figure S6. Biosafety and biocompatibility of nanomaterials. a** and **b,** Cytotoxicity in cultured neurons assessed using a PI uptake assay after AMF application. Merged fluorescence images show Fluo-4 (green) and PI (red) staining in the BTO_100_/MND_250_ group (**a**) and BTO_100_/HND_250_ group (**b**). **c,** Quantification of dead cell ratio calculated as the percentage of PI-positive neurons among Fluo-4-stained cells (n = 10). **d** to **f**, TEM images of cultured neurons treated with BTO_100_/MND_250_ for 0 (**d**), 3 (**e**), and 7 days (**f**). Left, A lower magnification image. Middle and Right, The magnified images from the square areas in Left. **g** and **h,** Cytotoxicity in HEK293T cells assessed using a Calcein-AM/PI live/dead assay after AMF application. Merged fluorescence images show Calcein-AM (green) and PI (red) staining in the BTO_100_/MND_250_ group (e) and BTO_100_/HND_250_ group (e). **i,** Quantification of dead cell ratio calculated as the percentage of PI-positive cells among total cells (n = 10).


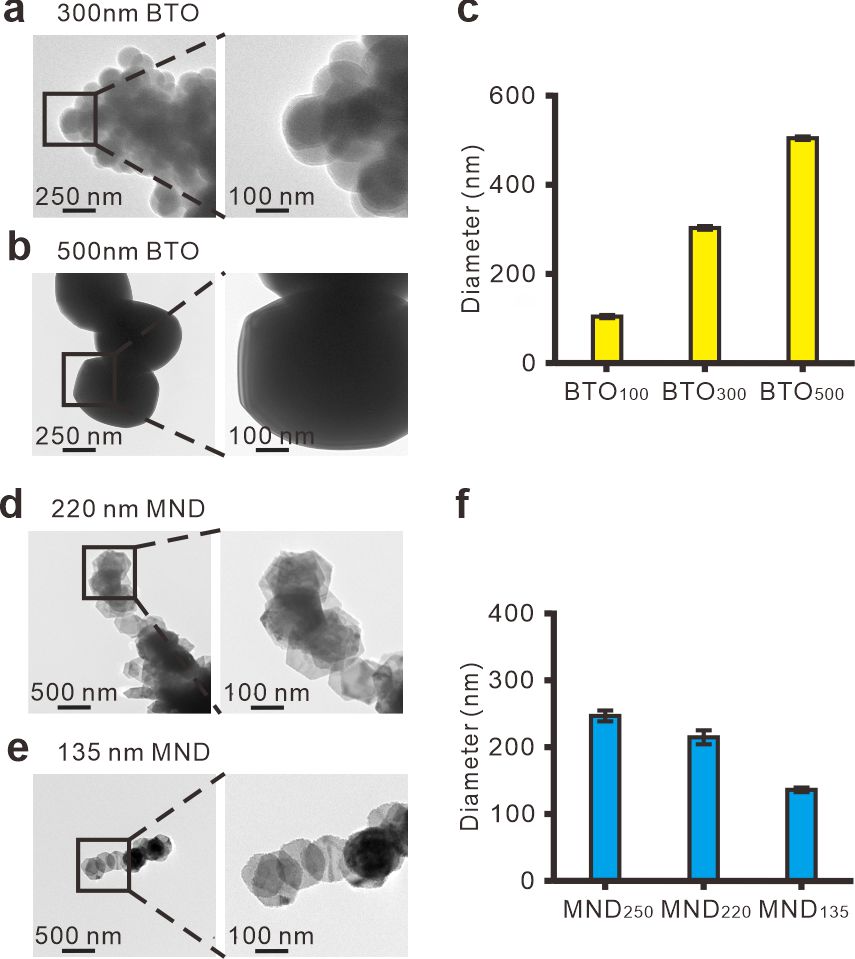


**Figure S7. MagTIES with different sizes of BTOs and MND**s. **a**, TEM images of BTO_300_. **b,** TEM images of BTO_500_. **c**, Diameters of BTOs. **d**, TEM images of MND_220_. **e**, TEM images of MND_135_. **f**, Diameters of MNDs.


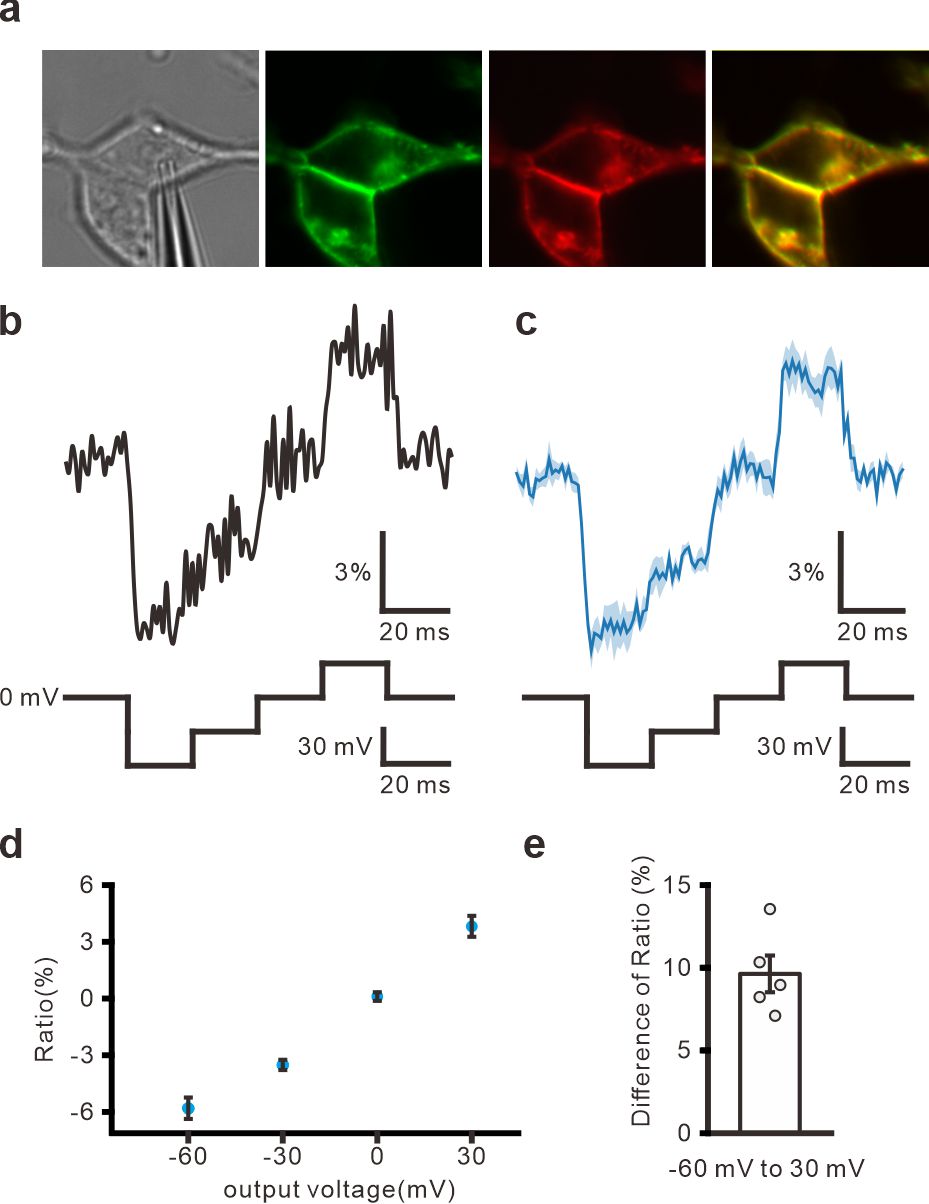


**Figure S8. Calibration of the Di-8-ANEPPS with whole-cell path-clamp electrophysiology on HEK293T cell. a,** A whole-cell recording on a HEK293T cell with Di-8-ANEPPS. Left to right, bright field, green fluorescence, red fluorescence, and merged image. **b,** Top, a representative voltage imaging trace when holding the membrane potential with voltage-clamp. Bottom, the protocol of voltage-clamp. **c,** Top, the average voltage imaging trace when holding the membrane potential with voltage-clamp (n = 5). Light blue area, s.e.m. of the average voltage imaging trace. Bottom, the protocol of voltage-clamp. **d,** The averaged green/red ratio at different holding potentials related to the baseline. **e,** the difference of green/red ratio at -60 mV and 30 mV (n = 5).


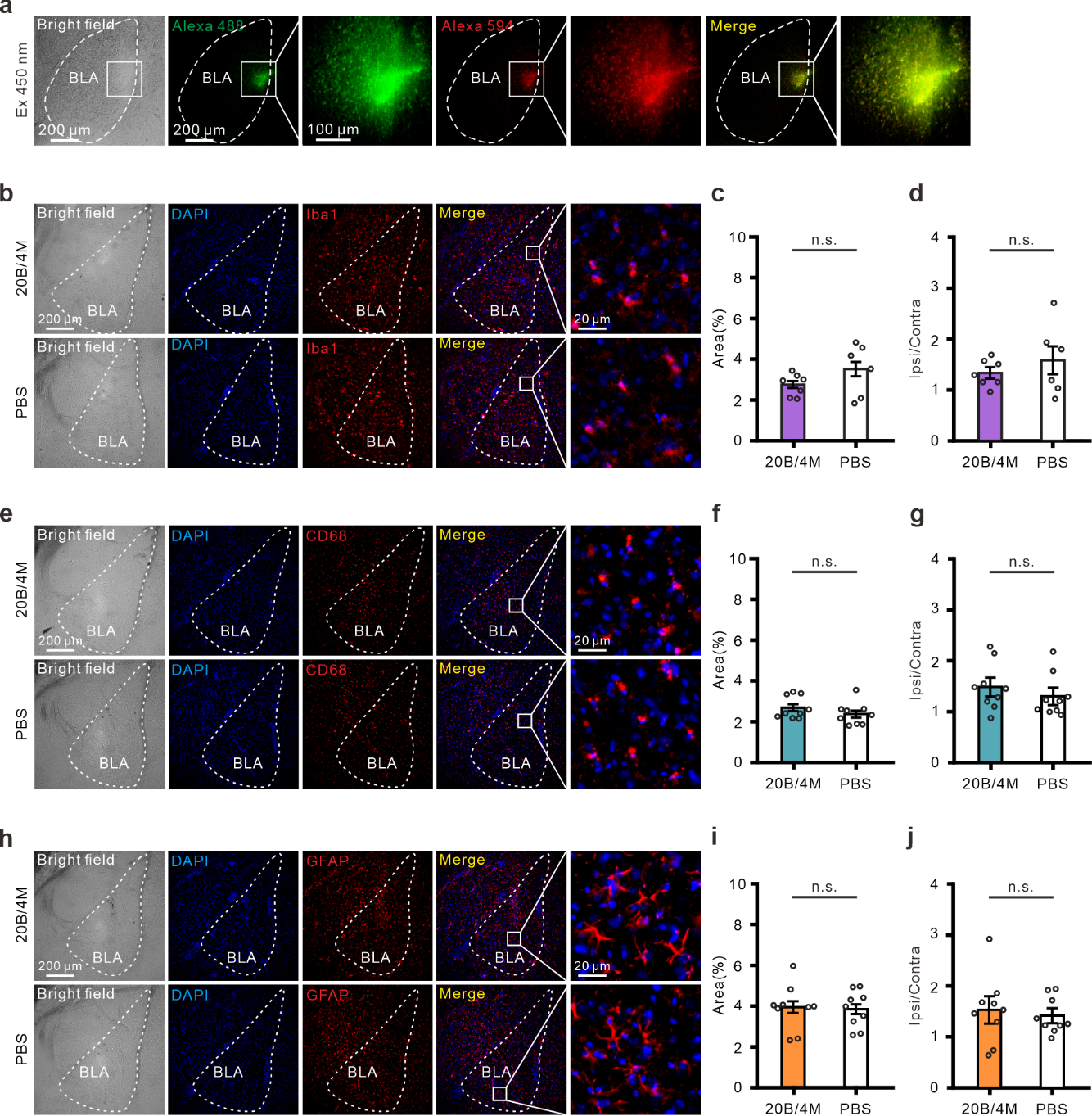


**Figure S9. Localization of nanomaterials and biocompatibility assessment in the basolateral amygdala (BLA). a,** Representative images showing the distribution of Alexa-488-conjugated BTO_100_ and Alexa-594-conjugated MND_250_ in the BLA. Fluorescence was recorded using 450 nm excitation to excite Alexa-488 on BTO, with detection of Alexa-594 emission indicating close proximity of BTO and MND nanoparticles. **b,** The fluorescence imaging of immunostaining of Iba1. Top, ipsilateral amygdala of mice injected with 20B/4M. Bottom, ipsilateral amygdala of mice injected with PBS. Left to right, bright field, DAPI, Iba1, merged image, and enlarged merged image. **c,** The Iba1 expression area in ipsilateral amygdala of 20B/4M (n = 7) and PBS group (n = 6). **d,** The ratio of Iba1 expression area in ipsilateral and contralateral amygdala of 20B/4M and PBS group. **e,** The fluorescence imaging of immunostaining of CD68. Top, ipsilateral amygdala of mice injected with 20B/4M. Bottom, ipsilateral amygdala of mice injected with PBS. Left to right, bright field, DAPI, CD68, merged image, and enlarged merged image. **f,** The CD68 expression area in ipsilateral amygdala of 20B/4M (n =9) and PBS group (n = 9). **g,** The ratio of CD68 expression area in ipsilateral and contralateral amygdala of 20B/4M and PBS group. **h,** The fluorescence imaging of immunostaining of GFAP. Top, ipsilateral amygdala of mice injected with 20B/4M. Bottom, ipsilateral amygdala of mice injected with PBS. Left to right, bright field, DAPI, GFAP, merged image, and enlarged merged image. **i,** The GFAP expression area in ipsilateral amygdala of 20B/4M (n = 9) and PBS group (n = 9). **j,** The ratio of GFAP expression area in ipsilateral and contralateral amygdala of 20B/4M and PBS group.


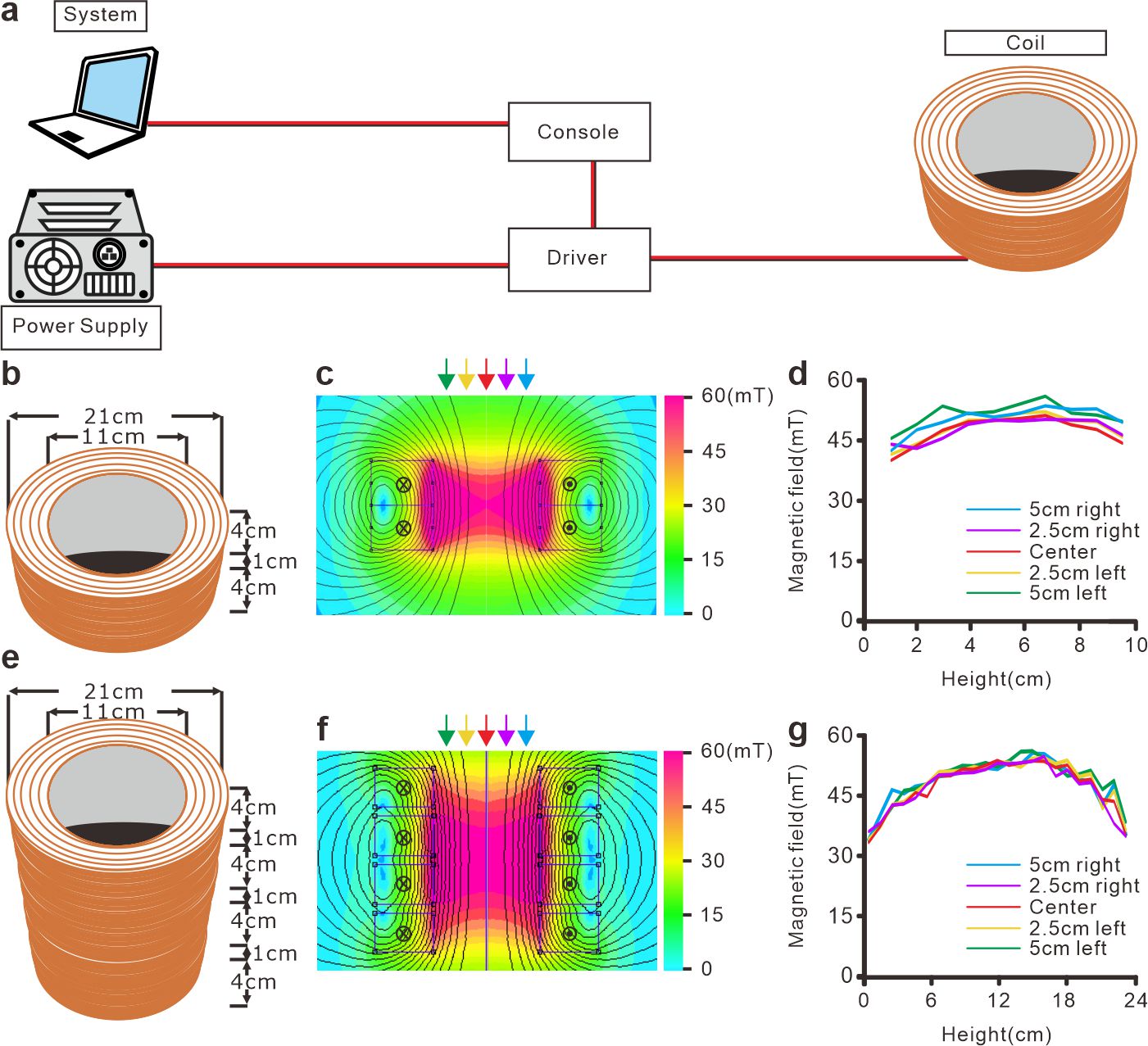


**Figure S10. Magnetic apparatus for *in vivo* experiments. a,** Scheme of magnetic setup for *in vivo* experiments. **b,** Dimension of magnetic coils for c-fos experiment. **c,** Heatmap of FEMM simulation for magnetic field distribution from coils designed in (**b**). **d,** Measured magnetic intensity from the coil by Gaussmeter. The measurement was vertically along the arrow indicated location in (**c**). **e,** Dimension of magnetic coils for fiber photometry experiment. **f,** Heatmap of FEMM simulation for magnetic field distribution from coils designed in (**e**). **g,** Measured magnetic intensity from the coil by Gaussmeter. The measurement was vertically along the arrow indicated location in (**f**).


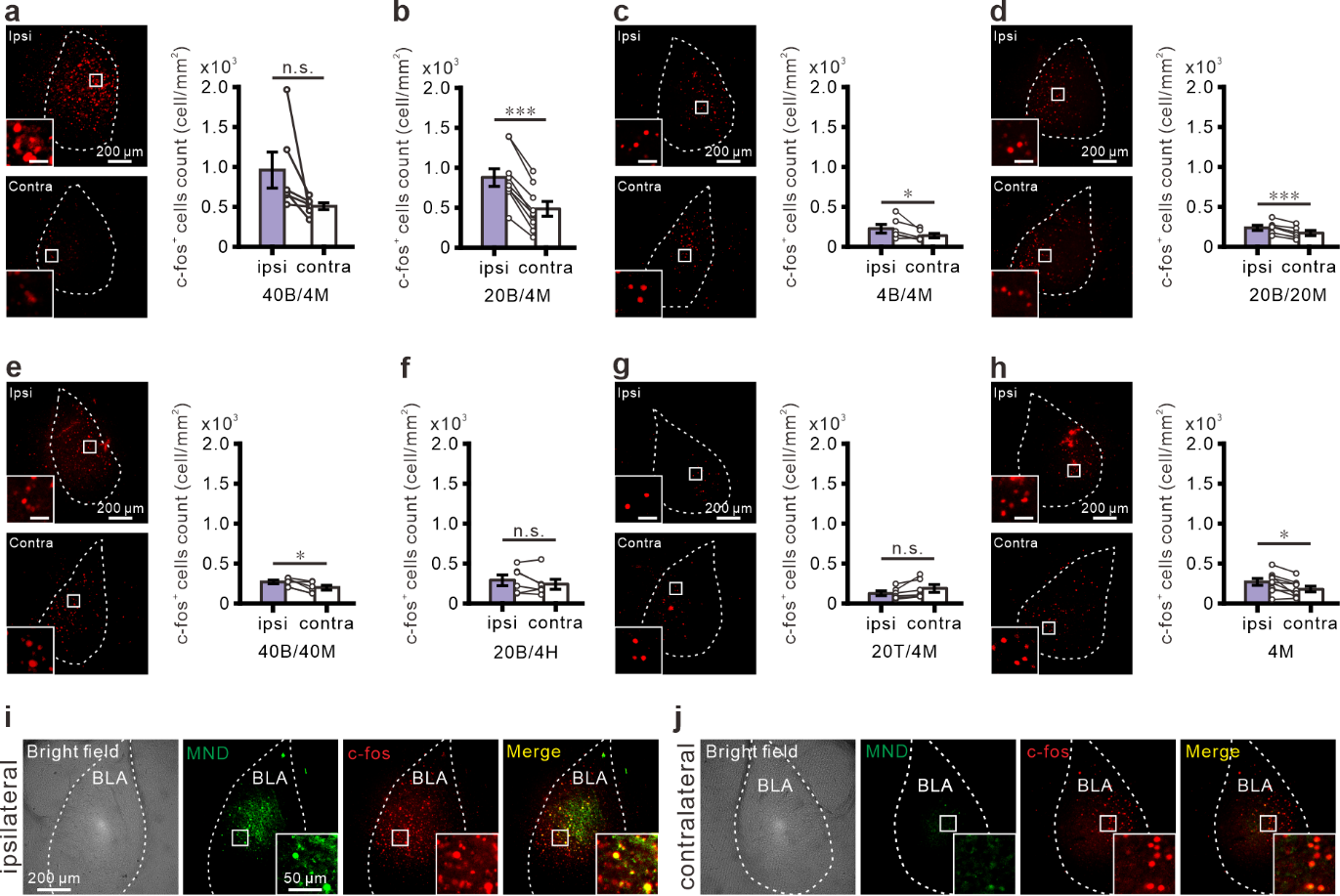


**Figure S11. MagTIES-induced c-fos expression in each hemisphere. a,** The c-fos expression in each hemisphere of 40B/4M group (n = 6). Left, immunostaining of c-fos in the ipsilateral (top) and contralateral (bottom) of basolateral amygdala (BLA). Inset, The magnified image of c-fos expression cell. Scale bar, 40 μm. Dashed lines, BLA. Square, magnified area. Right, Quantification c-fos expression level in both BLA. **b**, Right, Quantification c-fos expression level in both BLA of 20B/4M group (n = 9). ***p < 0.001, paired t-test. **c** to **e,** The c-fos expression in each hemisphere of 4B/4M (**c**, n = 6; *p = 0.048, paired t-test), 20B/20M (**d**, n = 6; ***p < 0.001, paired t-test), and 40B/40M group (**e**, n = 6; *p = 0.016, paired t-test). Left, immunostaining of c-fos in the ipsilateral (top) and contralateral (bottom) of basolateral amygdala (BLA). Inset, The magnified image of c-fos expression cell. Scale bar, 40 μm. Dashed lines, BLA. Square, magnified area. Right, Quantification c-fos expression level in both BLA. **f**, Right, Quantification c-fos expression level in both BLA of 20B/4H group (n = 6). **g** and **h,** The c-fos expression in each hemisphere of 20T/4M (**g**, n = 6) and 4M group (**h**, n = 9; *p = 0.011, paired t-test). Left, immunostaining of c-fos in the ipsilateral (top) and contralateral (bottom) of basolateral amygdala (BLA). Inset, The magnified image of c-fos expression cell. Scale bar, 40 μm. Dashed lines, BLA. Square, magnified area. Right, Quantification c-fos expression level in both BLA. **i** and **j,** Ipsilateral (i) and contralateral (j) amygdala slices from the 20B/4M groups. Left, Bright-field image. Middle-left, biotinylated MNDs stained by Streptavidin-FITC. Middle-right, immunostaining of c-fos. Right, merged image. Inset, magnified image.


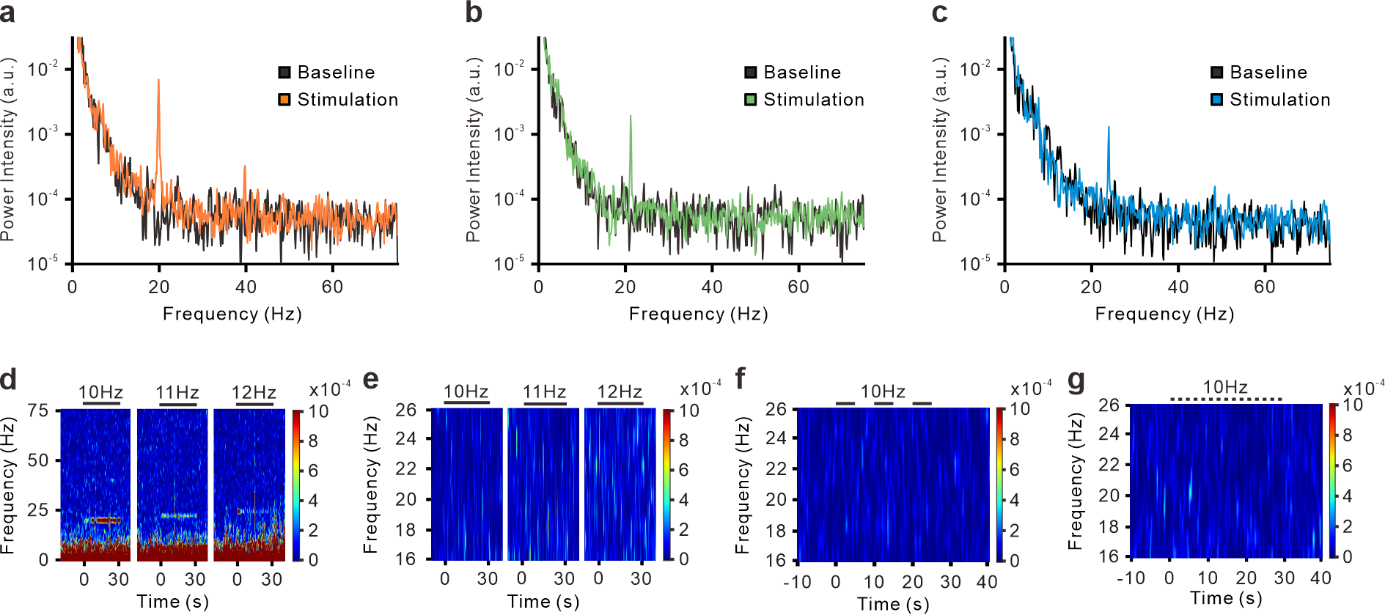


**Figure S12. Ca^2+^ responses by fiber photometry in BTO/MND or BTO/HND injected mice. a** to **c,** Power spectral density plots (logarithmic scale) of fluorescence signals in 20B/4M group during magnetic stimulation with 50 mT AMF at 10 Hz (a), 11 Hz (b), and 12 Hz (c) for 30 s. Black traces represent FFT calculated from the 20 s baseline period before stimulation. Colored traces represent FFT from the 30 s AMF stimulation period. **d,** Time–frequency spectrograms (0–75 Hz) of the 20B/4M group showing frequency-specific power increases during 50 mT AMF at 10, 11, and 12 Hz for 30 s. **e,** Spectrogram of MagTIES in 20B/4H group with 50mT AMF at 10, 11, and 12 Hz for 30 s. **f,** Spectrogram of MagTIES in 20B/4H group with 5 s AMF at 10 Hz for 3 times with 5 s intervals. **g**, Spectrogram of MagTIES in 20B/4H group with 1 s AMF at 10 Hz for 15 times with 1 s intervals.
